# Increased *Brucella abortus* asRNA_0067 expression under intraphagocytic stressors is associated with enhanced *virB2* transcription

**DOI:** 10.1007/s00203-024-03984-8

**Published:** 2024-05-31

**Authors:** Adrian Muñoz-Bucio, Beatriz Arellano-Reynoso, Félix J. Sangari, Rodrigo Sieira, Patricia Thébault, Clara Espitia, Juan M. García Lobo, Asunción Seoane, Francisco Suárez-Güemes

**Affiliations:** 1 Facultad de Medicina Veterinaria y Zootecnia, Departamento de Microbiología e Inmunología. Circuito Exterior S/N, Universidad Nacional Autónoma de México, CDMX, Ciudad Universitaria, Coyoacán, 04510 Mexico; 2 Instituto de Biomedicina y Biotecnología de Cantabria (IBBTEC), Universidad de Cantabria-CSIC-SODERCAN, C. Albert Einstein 22, 39011 Santander, Cantabria Spain; 3 Fundación Instituto Leloir-IIBBA CONICET, Av. Patricias Argentinas 435CABA, CP. 1405 Buenos Aires Argentina, Argentina; 4 Laboratoire Bordelais de Recherche en Informatique (LaBRI), UMR 5800, CNRS, Bordeaux INP, Université de Bordeaux, 33400 Talence, France; 5 Departamento de Inmunología, Instituto de Investigaciones Biomédicas, Universidad Nacional Autónoma de México MX, CDMX, Circuito Escolar 33, Ciudad Universitaria, Coyoacán, 04510 Mexico

**Keywords:** *Brucella abortus*, Intraphagocytic adaptation, Stressors, *virB2*, Antisense RNAs

## Abstract

Intracellular pathogens like *Brucella* face challenges during the intraphagocytic adaptation phase, where the modulation of gene expression plays an essential role in taking advantage of stressors to persist inside the host cell. This study aims to explore the expression of antisense *virB2* RNA strand and related genes under intracellular simulation media. Sense and antisense *virB2* RNA strands increased expression when nutrient deprivation and acidification were higher, being starvation more determinative. Meanwhile, *bspB*, one of the T4SS effector genes, exhibited the highest expression during the exposition to pH 4.5 and nutrient abundance. Based on RNA-seq analysis and RACE data, we constructed a regional map depicting the 5' and 3' ends of *virB2* and the cis-encoded asRNA_0067. Without affecting the CDS or a possible autonomous RBS, we generate the deletion mutant ΔasRNA_0067, significantly reducing *virB2* mRNA expression and survival rate. These results suggest that the antisense asRNA_0067 expression is promoted under exposure to the intraphagocytic adaptation phase stressors, and its deletion is associated with a lower transcription of the *virB2* gene. Our findings illuminate the significance of these RNA strands in modulating the survival strategy of *Brucella* within the host and emphasize the role of nutrient deprivation in gene expression.

**Supplementary Information:**

The online version contains supplementary material available at 10.1007/s00203-024-03984-8.

## Introduction

*Brucella abortus*, a Gram-negative facultative intracellular pathogen, is the etiological agent of brucellosis, a severe and costly disease affecting various hosts, including domestic animals, wildlife, and humans (Pappas et al. 2006). Upon infection, *Brucella* relies on its ability to resist and take advantage of the hostile environment of the host phagocytic cells to establish a chronic infection (Criscitiello et al. 2013; Porte et al. 1999). This complex intracellular passage involves multiple stages, with the adaptation phase being crucial for the later persistence and evasion of the host immune system. *Brucella* encounters a spectrum of intraphagocytic stressors during this phase, including acidic pH and nutrient scarcity (Sieira 2013; Köhler et al. 2002). This pathogen has developed an outstanding ability to sense its environment and respond through a network of genomic regulation, such as the regulatory circuit of the BvrS-R/VjbR/VirB sensor-effector system. This circuit effectively detects nutrient depletion and acidification of the environment during the late endosome phase. BvrS, as the sensor in the two-component system, perceives the environmental changes and transmits a phosphorylating signal to the effector BvrR. The latter functions as a promoter for the *vjbR* and *virB* operon expression. Between these two components, a loop of positive mutual feedback exists, resulting in substantial transcription of the entire *virB* operon along with its effectors (Altamirano-Silva et al. 2018; Wang et al. 2009). The *virB* operon is a gene cluster encoding the Type IV Secretion System (T4SS), which enables direct delivery of effectors to the host cell cytoplasm to modulate the *Brucella*-containing vacuole (BCV) formation and intracellular trafficking. An essential *virB* operon component, the *virB2* gene, encodes the pili-like structure vital for T4SS function, allowing *Brucella* to deliver effectors into the host cell cytoplasm, manipulating signaling and ensuring survival (Celli 2020; Ke et al. 2015; O'Callaghan et al. 1999).

Non-coding RNAs (ncRNAs) regulate gene expression in bacteria (Waters and Storz 2009), influencing virulence factors, metabolism, and stress response genes during intracellular infection (Caswell et al. 2012; Hoe et al. 2013). ncRNAs are classified based on location: Trans-encoded RNAs are outside the regulated gene loci. Cis-encoded RNAs, like antisense RNAs (asRNAs) (Watkins and Arya 2019), are perfectly complementary to the target mRNA and aid in adapting to environmental changes (Lejars et al. 2019; Millar and Raghavan 2021). For instance, AS-fliR acts as an antisense RNA to the flagellar assembly *fliPQR* operon in *Salmonella enterica*, impacting swarming motility upon mutation (Wang and Harshey 2009). In the context of *Brucella*, the presence of ncRNAs inside the *virB* operon remains unexplored.

This study aimed to characterize the double-stranded transcriptome of *Brucella abortus* under Intraphagocytic Simulation Media (ISM) during the adaptation phase of the infection. Through RNA-seq analysis of available databases and expression profiling with RT-qPCR, we examined the differential expression of *virB2* and its antisense RNA, designated as asRNA_0067. This analysis helped to elucidate the role of asRNAs in the fine regulation of *virB2* expression, a critical component of the T4SS, and other essential genes involved in intracellular adaptation.

## Materials and methods

### Strains and media

In this study, we used the field pathogenic strain *Brucella abortus* 2308W (2308W) and two RNA mutants, the ΔasRNA_0067 and ΔRNA_0069. All strains were grown in Brucella broth (BB) (BD BBL, Franklin Lakes, NJ, USA) at 37° C, 150 rpm inside a BSL-3 laboratory. We performed growth kinetics for each strain in BB at 37 °C and 150 rpm to establish baseline behavior, enabling subsequent comparison of differences in adaptability to intraphagocytic stress factors.

We reviewed the scientific literature on the stressors *Brucella* is exposed to during the adaptive phase of intraphagocytic trafficking, in which the *virB* operon plays an essential role. Nutrient deprivation and acidic pH are the most critical factors during the adaptive phase. We relied on the minimal medium GEM (Wang et al. 2015) with modifications by reducing the glucose concentration to first generate the modified GEM (mGEM) and then to establish the Intraphagocytic Simulation Media (ISMs) ISM_A1 (pH 4.5) and ISM_A2 (pH 5). We also used the minimal medium MM1 pH 5.5 as a reference because we observed both RNAs 0067 and 0069 in an RNA-seq analysis database of *B. abortus* under these stressors (Kleinman et al. 2017; Sieira et al. 2010). ISM_R at pH 6.5 was derived from MM1 to mimic the initial replication phase. BB at pH 7 served as the non-stress control for differential expression analysis by RT-qPCR. Standardizing the pH to 4.5 across all media allowed us to assess the impact of nutrient deprivation. Consequently, BB and ISM_R at pH 4.5 were compared with ISM_A1 (pH 4.5). Table 1 outlines the composition of each ISM.

**Table 1:** Intraphagocytic Simulation Media (ISMs) based on *Brucella* intraphagocytic conditions

Intraphagocytic phase	ISM	pH	Reagents	Modified from
**Adaptative phase (A)** - Acidic pH - Nutrient deprivation 4 h **[a]**	**ISM_A1**	pH 4.5 ** [a]**	2 g/L Glucose **[f]** 2 g/L Citric acid 10 g/L Dibasic Potassium Phosphate 3.5 g/L Sodium ammonium phosphate 0.096 g/L Magnesium sulfate	GEM ** [g]**
	**ISM_A2**	pH 5.0 **[c,d,e]**		
**Replicative phase (R)** - Neutral pH - Nutrient acquisition 4 h **[b]**	**ISM_R**	pH 6.5 ** [c,e]**	1 g/L Yeast extract 4.4 g/L Monobasic potassium phosphate 10.5 g/L Dibasic potassium phosphate	MM1 ** [h]**

Brucella broth (BB) at pH 7 was used for bacterial growth and as the non-stress control for differential expression analysis. Additionally, BB and ISM_R at pH 4.5 were employed to evaluate the impact of nutrient deprivation.

ISM: Intraphagocytic Simulation Medium, ISM_A: ISM_Adaptative, ISM_R: ISM_Replicative

^**a**^Porte et al. 1999,

^**b**^de Bolle et al. 2015,

^**c**^Bellaire et al. 2005,

^**d**^Repnik et al. 2013,

^**e**^Maxfield and Yamashiro 1987,

^**f**^Koobotse et al. 2020,

^**g**^Wang et al. 2015,

^**h**^Sieira et al. 2010

### Bioinformatics data analysis

We employed a comprehensive RNA-seq analysis using a publicly accessible database to explore the transcriptional landscape of *B. abortus* 2308W during acidification and nutrient deprivation, including both mRNAs and ncRNAs. High-quality reads were downloaded from the Gene Expression Omnibus database (https://www.ncbi.nlm.nih.gov/geo/) under accession number GSE95722 (Kleinman et al. 2017). Both stranded counts visualization within the region of *virB* operon and quantification of gene expression levels were performed using the software IGV (http://broadinstitute.org/igv) to identify differentially expressed genes (DEGs) between the parental 2308W strain and its *vjbR* deletion mutant.

To identify the interactions between ncRNAs and genes within the *virB* operon, its effectors, and other target genes, we conducted a predictive analysis of RNA-mRNA interactions using the IntaRNA server (Mann et al. 2017) (http://rna.informatik.uni-freiburg.de). Once predicted target mRNAs were obtained for each ncRNA, the subsequent step involved searching for functional annotation and genomic data of interest for each mRNA with the Gene NCBI database (https://www.ncbi.nlm.nih.gov/gene/) and KEGG Orthology (https://www.genome.jp/kegg/ko.html).

### RNA isolation

Total and small RNA from 3 mL of four-hour (lag phase) *Brucella* cultures were extracted and purified using 6 mL of Bacterial RNAprotect, 700 µL of QIAzol lysis reagent, and miRNeasy kit (QIAGEN Inc., Chatsworth, CA, USA), according to the manufacturer's guidelines. Lysozyme was used for bacterial cell digestion, and proteinase K was added when RNA isolation was made from complex media such as BB. DNase treatment with DNase I (QIAGEN) was carried out in all RNA isolations inside the column during the purification step according to the manufacturer’s guidelines. The presence of contaminating genomic DNA was tested by qPCR targeting the 16S rRNA gene.

### RT-qPCR

Reverse transcription of RNA extracted from the intraphagocytic stress assays was carried out with the Superscript III kit (Invitrogen, Carlsbad, CA, USA) using random hexamers and following the manufacturer’s instructions. The quantitative reverse transcriptase PCR (RT-qPCR) was carried out using TB Green (Takara Bio Inc., Tokyo, Japan) to validate the presence and quantify non-coding RNAs and adaptation-related genes. All primers were designed with the PrimerPlus3 server (https://www.primer3plus.com/index.html) and are listed in Supplementary Information 1. The qPCR parameters were as follows: pre-incubation at 95 °C for 10 min; amplification, 35 cycles of denaturation at 95 °C for 10 s, annealing at 62 °C for 20 s, and elongation at 72 °C for 25 s. The melting curves were obtained by subjecting the samples to 95 °C for 5 s, 62 °C for 1 min, and 95 °C for 1 s. We calculated the 2^−ΔΔCt^ (Livak and Schmittgen 2001) using 16S rRNA as the reference gene and the medium BB pH 7 as the non-stress control for the differential expression analysis. Experiments were conducted in duplicate with three independent replicates.

### Survival assays

First, each strain was cultured to log phase (20 h) in pH 7 at 37 °C and 150 rpm. We then measured the absorbance to homogenize the initial concentration to 0.020 optical densities (ODs) at 600 nm across all the strains exposed to the stress conditions described below. The effect of each factor was assayed by CFU counting and comparing the number before and after incubation under different conditions to obtain the survival rate. The initial CFU (0 h) was determined to be 100% of the survival rate. Total and small RNA was extracted from these experiments at 4 h post incubation for further expression analysis using the abovementioned method.

### RACE

The Rapid Amplification of cDNA Ends (RACE) technique was employed to characterize the 5' and 3' ends of asRNA_0067 and its antisense gene *virB2* in *B. abortus* 2308W. Total RNA was isolated using the abovementioned method, followed by polyadenylation using poly(A) polymerase (New England Biolabs, Ipswich, MA, USA) at 37 °C for 30 min and EDTA 10 mM to stop the reaction. Reverse transcription to obtain 5- and 3'-RACE-Ready, and PCR-RACE was carried out following the SMARTer RACE (Takara) instructions for prokaryotic RNA samples. The resulting RACE products were gel-purified and sequenced using nested gene-specific primers (NGSPs). GSPs and NGSPs for RACE characterization are described in Supplementary Information 1. Thermocycle for RACE PCR was programmed as follows: 5 cycles of 94 °C for 30 s, 72 °C for 3 min, 5 cycles of 94 °C for 30 s, 70 °C for 30 s, 72 °C for 3 min and 34 cycles of 94 °C for 30 s, 68 °C for 30 s and 72 °C for 3 min.

### Isothermal assembly deletion mutation

Deletion mutations of asRNA_0067 and RNA_0069 were performed using the isothermal assembly method (Gibson et al. 2009). Primers were designed for PCR amplification of upstream and downstream fragments of the sequences to be deleted for the RNA gene (Supplementary Information 2). The PCR was conducted with the following concentrations: 5× Phusion HF Buffer 4 µL, 0.2 mM dNTPs, 0.5 µM of each primer, Phusion DNA polymerase 0.4U (Phusion High-Fidelity DNA Polymerase, New England Biolabs Inc., Hertfordshire, UK), DNA (*B. abortus* 2308W) 17 ng, H_2_O up to 20 µL. The amplification program was as follows: 98 °C for 30 s, followed by 30 cycles: 98 °C for 10 s, 70 °C for 10 s, 72 °C for 20 s, concluding with a cycle at 72 °C for 5 min. Meanwhile, plasmid pDS132 (kindly donated by Dominique Schneider, University Joseph Fourier, Grenoble, France) (Philippe et al. 2004) was amplified with the same reagent concentrations mentioned above and 21 ng of pDS132 DNA, H_2_O up to 20 µL. The amplification program was also the same for the fragment, except that the 30 cycles consisted of 98 °C for 10 s and 72 °C for 2 min. Following amplification, the resultant fragment was purified using the GenElute PCR Clean-Up Kit (Sigma Aldrich, San Luis, MO, USA) and subjected to enzymatic digestion (Fast digest DpnI, Thermofisher Scientific Waltham, MA, USA) for 15 min at 37 °C, followed by another purification step. For the isothermal assembly technique, the fragment was combined at equimolar concentrations with the plasmid (150 ng) in a volume of 5 µL of ddH_2_O. This mixture was then added to 15 µL of the 5× Isothermal assembly premix, containing: 0.5 M Tris–HCL pH 7.5, 0.1 M MgCl_2_, 1 mM of each dNTP, DTT 50 mM, PEG 0.25 g/mL, 5 mM NAD, and 12U T5 exonuclease (Epicentre, Illumina, Madison, WI, USA). The assembly reaction was subjected to 50 °C for 1 h, followed by dialysis in ddH_2_O. Subsequently, *E. coli* β2163 (Demarre et al. 2005) was transformed with 1 µL of the assembly reaction. Finally, *B. abortus* 2308W was transformed by conjugation with *E. coli* β2163.

### Statistics

Differential expressions of transcripts and survival rates were evaluated using the T-Test on GraphPad 9 software (GraphPad Prism Software, Inc., La Jolla, CA, USA). The results represent means from at least three independent experiments, with standard deviations included. Statistical significance was determined at p-values < 0.05.

## Results

### Identification of two antisense RNAs to the *virB* operon of *Brucella abortus* 2308W expressed during adaptation to acidic pH and nutrient deprivation

**Fig. 1:**
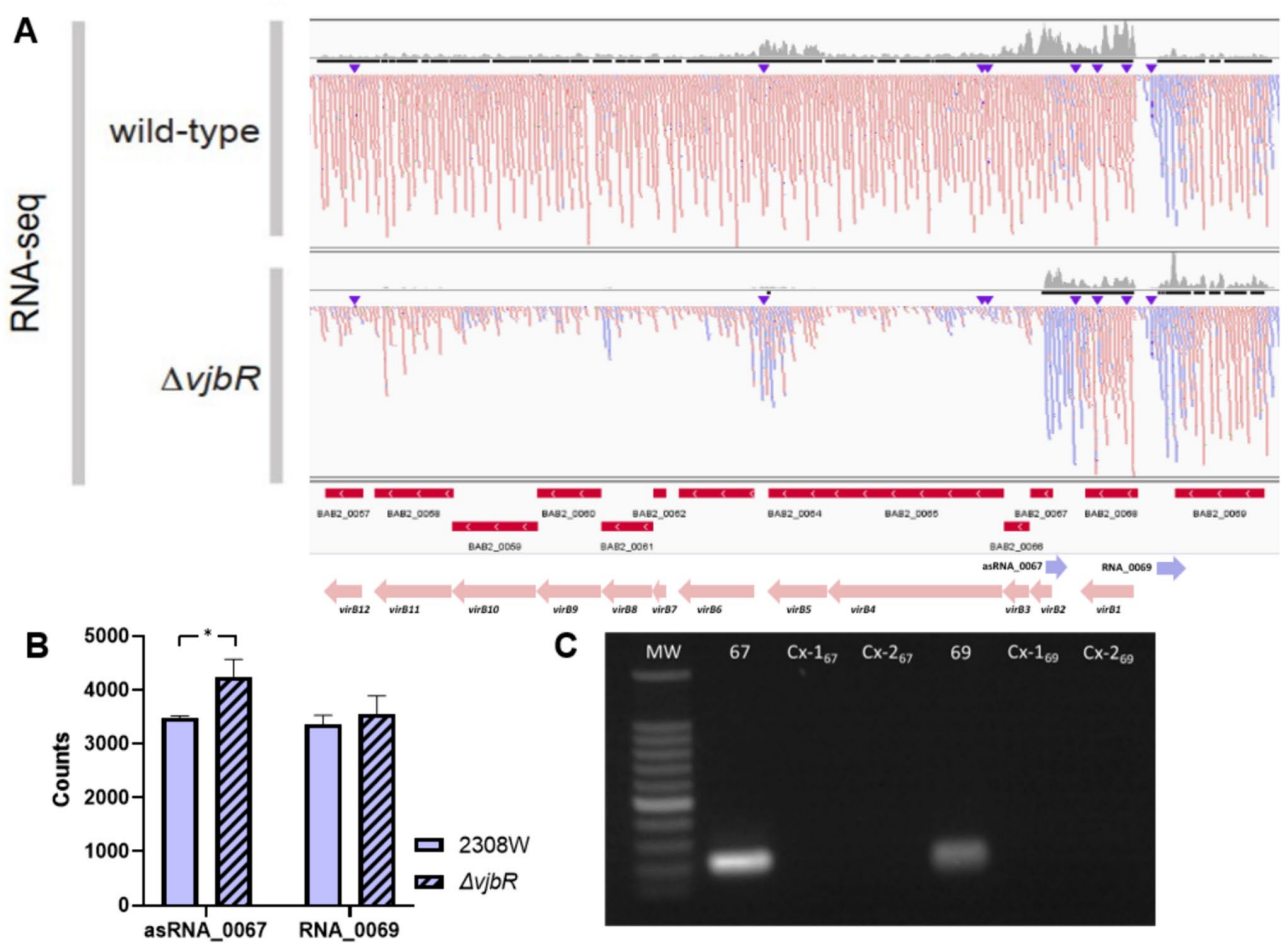
Detection and quantification of two antisense RNAs to the *virB* operon in a minimal nutrient medium at pH 5.5. RNA-seq analysis from an open access database of *B. abortus* 2308W in MM1 pH 5.5 reveals differences in *virB* operon and antisense RNA expression between the parental strain 2308W and its Δ*vjbR* knockout mutant, presented in a genetic map (**A**) and counts graph (**B**). Antisense transcripts on the positive strand are depicted in blue, while transcripts on the negative strand, housing the *virB* operon, are represented in red. The RNA-seq results and their significance are based on three independent replicates. The presence of cDNA from RNAs 0067 and 0069 is validated through agarose gel electrophoresis of RT-qPCR products (**C**). MW: 1 kb plus molecular weight marker, 67: asRNA_0067, 69: RNA_0069, Cx-1_67_ and _69_: RNA without gDNA, Cx-2_67_ and _69_: no template

To uncover regulatory RNA elements within operon *virB* of *Brucella abortus*, we utilized publicly accessible databases of RNA-seq analysis. Specifically, we examined the transcriptional landscape of *B. abortus* 2308W under conditions of pH 5.5 within the minimal nutrient medium MM1 (Kleinman et al. 2017). This analysis unveiled the presence of two non-coding RNAs (ncRNAs) residing on the complementary strand adjacent to the initial segment of the *virB* operon. The RNA_0069 is located in the antisense strand between the genes BAB2_0069 and *virB1*. Meanwhile, the asRNA_0067 is situated antisense and between the *virB1* and *virB2* genes, exhibiting high sequence complementarity with the *virB2* gene, leading to its designation as asRNA_0067 **(**Fig. 1A**)**. This RNA-seq analysis assessed the impact of a mutation in the quorum sensing-associated gene, *vjbR*. While conducting an expression count analysis of both RNAs, a slight yet statistically significant increase in asRNA_0067 abundance was observed in the mutant strain compared to the parental strain **(**Fig. 1B**)**.

To validate the presence of non-coding RNAs _0067 and 0069, we performed RNA extraction and RT-PCR from the assay for adaptation to stressors with MM1 minimal medium at pH 5.5 used for analysis from RNA-seq. As shown in Fig. 1C, two bands of approximately 100 bp in the electrophoresis represent the presence of cDNA of the non-coding antisense RNAs of the *virB* operon from this study. For RT-PCR, two controls were added for each RNA. Control 1 (Cx-1) contains the extracted RNA and is essential for proving the absence of genomic DNA (gDNA) that might have remained a residue if DNase treatment had failed during RNA extraction. Control 2 (Cx-2) contains no template to ensure that reagents are not contaminated and primers are correctly designed.

### asRNA_0067 predictably interacts with *virB2* with a high p-value and low FDR

The analysis of predicted RNA-RNA interactions revealed significant associations between asRNA_0067 and essential *Brucella* genes related to the T4SS. Notably, asRNA_0067 demonstrated a robust Watson–Crick base pairing interaction of 90 nucleotides with its antisense gene *virB2* (BAB2_0067), a critical component of the T4SS pili-like structure, exhibiting an optimal p-value and false discovery rate (FDR). Additionally, asRNA_0067 exhibited a significant interaction with the T4SS effector gene associated with rBCV biogenesis, *bspB* (BAB1_0712), showing an optimal p-value but a high FDR. Interactions with both *virB2* and *bspB* are localized within the same region of asRNA_0067. Predicted interactions also involved RNA_0069 with BAB2_0233, a TonB-dependent copper receptor gene, and BAB1_1830, a LemA family protein with an unknown function in *Brucella* but belonging to the Cluster of Orthology Genes (COG) T associated with signal transduction mechanisms (Table 2).

**Table 2:** RNA-mRNA predicted interactions in IntaRNA

Target	Query	Start	End	Energy of interaction	p-value	FDR	Annotation
BAB_RS26680 (BAB2_0067)	**asRNA_0067**	150	239	−137.48	0	0	***virB2*** (T4SS pili-like structure)
BAB_RS19325 (BAB1_0712)		213	249	−15.57	0.023718	0.942	***bspB*** (rBCV biogenesis T4SS effector)
BAB_RS27485 (BAB2_0233)	**RNA_0069**	165	198	−24.21	9.70E-06	0.031	**TonB-dependent copper receptor gene** (Copper homeostasis; COG: P)
BAB_RS24625 (BAB1_1830)		48	112	−21.19	0.000211	0.203	**LemA family protein** (Unknown function; COG: T)

### asRNA_0067 displays higher expression than RNA_0069 in modified GEM minimal medium

**Fig. 2:**
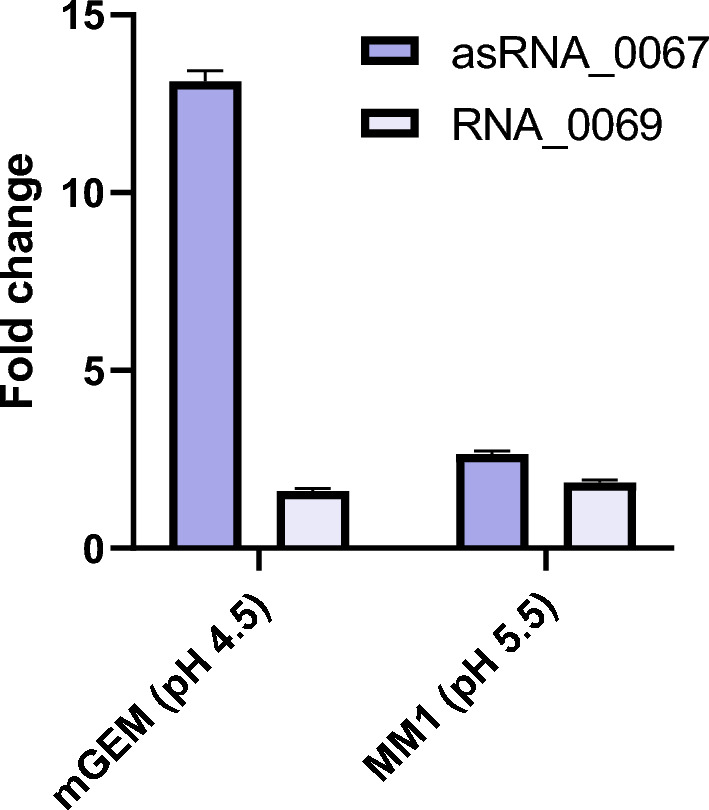
Differential expression analysis of asRNA_0067 and RNA_0069 comparing two media with intraphagocytic stressors and the particular medium for *Brucella* at 4 h of incubation. Fold change in expression of each RNA was observed in the minimal media mGEM pH 4.5 and MM1 pH 5.5 compared to BB pH 7, with a more significant difference seen for asRNA_0067 in the mGEM pH 4.5 medium*.* mGEM: modified GEM minimal medium

Differential expression analysis of asRNA_0067 and RNA_0069 was performed by comparing their quantification in BB pH 7 with two intraphagocytic simulation media with acidic pH and different levels of nutrient deprivation, mGEM and MM1. The expression of asRNA_0067 increases 13.1-fold in mGEM pH 4.5 compared to the expression obtained in BB pH 7, a nutrient-rich medium specific for this pathogen. In contrast to the 2.6-fold increase in MM1 pH 5.5 compared with BB pH 7, RNA_0069 exhibits minimal differential expression, 1.6 and 1.8 in mGEM pH 4.5 and MM1 pH 5.5, respectively **(**Fig. 2**)**. Consequently, the further analysis continued with asRNA_0067 only, as its expression is upregulated when *Brucella* is exposed to intraphagocytic stressors and its association with the predicted interacting genes related to the *virB* operon.

### pH and nutrient deprivation are critical factors for gene regulation of *Brucella* adaptation inside phagocytes

After a comprehensive search of the pH values, exposure time, and degree of nutrient deprivation, we formulated the optimal intraphagocytic simulation media mainly during the adaptation phase. This stage represents the period of maximum intensity in terms of acidic and nutritional stress. The information reported differs in that the pH drops to values of approximately 4.5 (Porte et al. 1999) and 5 (Bellaire et al. 2005; Maxfield and Yamashiro 1987; Repnik et al. 2013), so we evaluated both values in the ISM_Adaptative1 (ISM_A1) and ISM_A2, respectively. The time in which *Brucella* resists the acidification begins at the first hour post-infection, lasting approximately 4 h until the fifth-hour post-infection (Porte et al. 1999), in which the peak of expression of the *virB* operon is observed (Sieira 2013). The information about the degree of nutrient deprivation we found was practically null, so we could only reduce the amount of glucose in the GEM minimal medium from 20 to 2 g/L (Koobotse et al. 2020). To compare the results of the adaptation phase, we designed a medium simulating intracellular stress during the early nutrition and replication phase, the ISM_Replicative (ISM_R) at pH 6.5. Literature reports indicate that some *Brucella* cells reach the endoplasmic reticulum (ER) 2 h post-infection (Arellano-Reynoso et al. 2005; Celli et al. 2003), where they may come into contact with nutrients from these organelles, even as acidification continues. Consequently, we tested ISM_R at pH 4.5 and Brucella Broth at pH 4.5. In Table 1, we summarized the selected media with their respective pH values to simulate the abovementioned phases during intracellular trafficking.

### asRNA_0067 and *virB2* expression increased when mainly nutrient deprivation and also acidity were higher

**Fig. 3:**
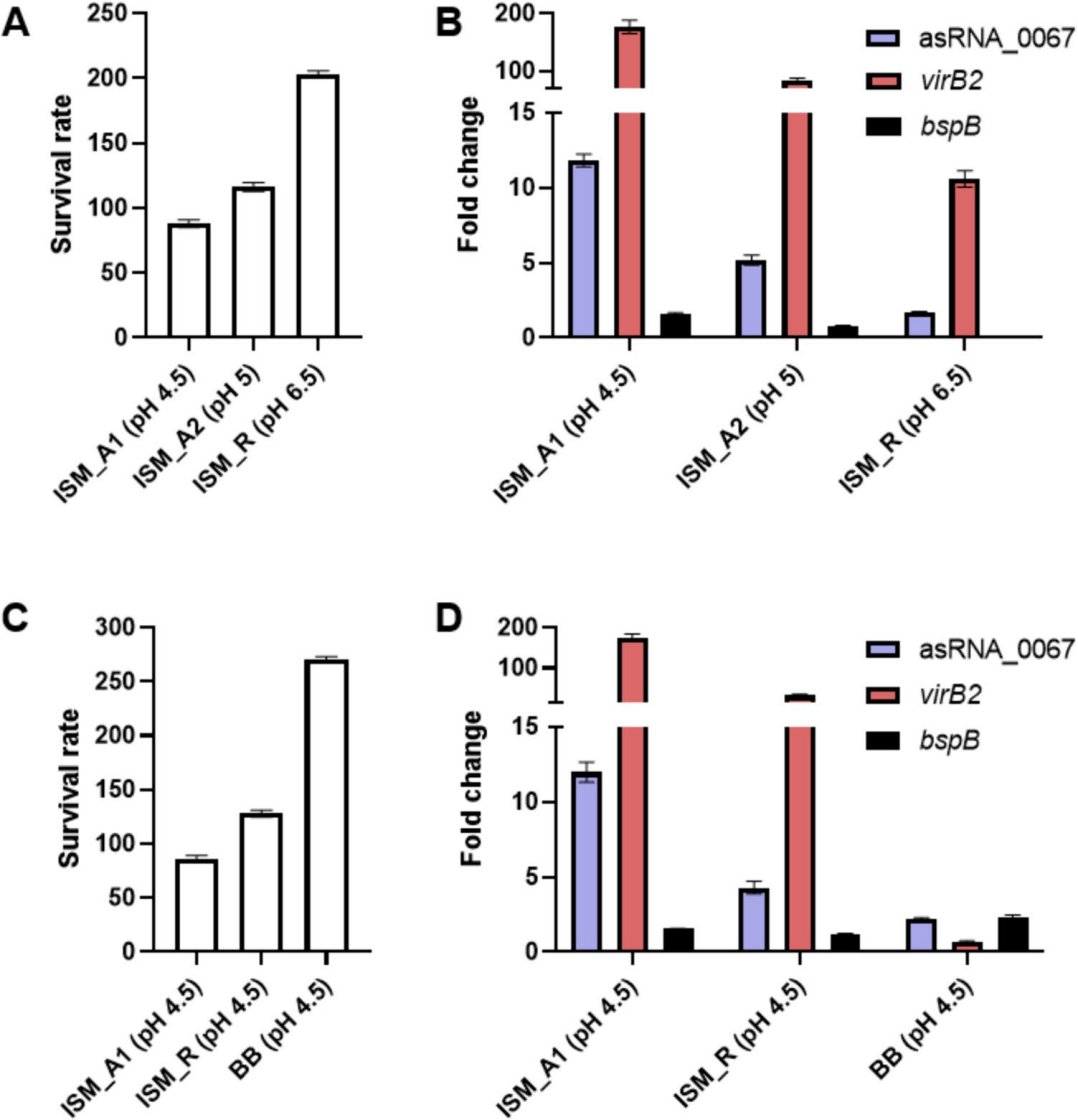
Survival and gene expression analysis of *Brucella abortus* 2308W under different nutrient deprivation and acidity levels. Differential transcript expression and survival rate at intracellular simulation media (**A–B**) or pH-standardized media (**C–D**) were calculated as the difference in CFU between 0 and 4 h of incubation, and fold change levels were compared to samples cultured in BB at pH 7. Values are shown as means of 3 experiments ± standard deviations. ISM: Intraphagocytic Simulation Medium; ISM_A: ISM_Adaptative; ISM_R: ISM_Replicative. BB: Brucella broth

Under the chosen conditions for Intraphagocytic Simulation Media (ISM), we conducted the adaptation assays to measure the survival rate and differential gene expression in *B. abortus* 2308W. In ISM_R, there was a remarkable 203% survival rate, indicating a two-fold replication. However, the expression of asRNA_0067 was the lowest among all the media evaluated. On the other hand, ISM_A1 (pH 4.5) provided the most hostile conditions, decreasing the survival rate to 88% and simultaneously inducing a higher expression of asRNA_0067 and *virB2*. In the same minimal medium at pH of 5 (ISM_A2), which is only 0.5 units more alkaline, we observed a nearly 50% reduction in expression compared to the medium with a pH of 4.5 and a 28% higher survival rate. The gene *bspB,* which encodes an effector of the T4SS, exhibited more expression as the pH was lower, showing a 1.6-fold change compared to BB (rich medium at pH 7). Together these results show that with higher magnitudes of intraphagocytic stressors, there is a lower survival rate and higher expression of asRNA_0067 and *virB2* (Fig. 3A, B).

To elucidate the impact of nutrient deprivation, we standardized the pH to 4.5 across all media used thus far. In BB pH 4.5, the highest survival rate was observed, accompanied by the lowest expression of *virB2* among all the assessed media. In ISM_R at pH 4.5, with an intermediate nutrient level, a survival rate of 128% was recorded, along with differential expression levels of *virB2* and asRNA_0067 at 31-fold and 4.1-fold higher, respectively. When comparing this medium to its pH 6.5 counterpart, a 75% decrease in survival rate was evident, coupled with a 21.5-fold and 2.5-fold increase in *virB2* and asRNA_0067 expressions, respectively (Supplementary Information 3). Lastly, the highest expression of the effector gene *bspB* among all media was observed in BB pH 4.5, with a 2.3-fold increase (Fig. 3C, D).

### asRNA_0067 overlaps with the coding sequence of its antisense gene *virB2* by 164 nucleotides

**Fig. 4:**
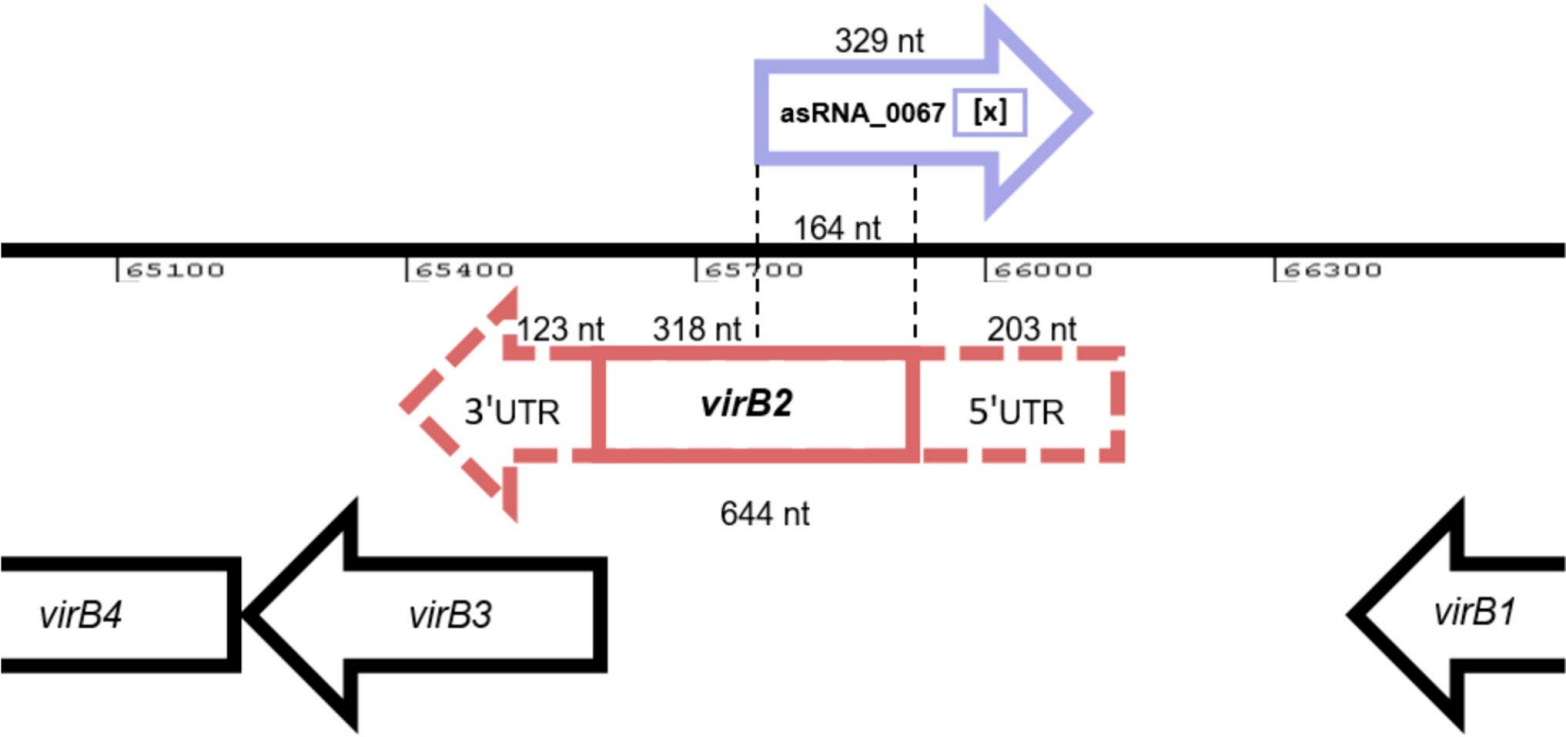
Double-stranded regional genetic map of *virB* operon and determination of the 5' and 3' ends of asRNA_0067 and *virB2*. Genetic map depicting the 164-nucleotide overlap between asRNA_0067 and *virB2* CDS in *B. abortus* 2308W. Characterization and RACE analysis revealed the complete 329-nucleotide sequence of asRNA_0067. The *virB2* gene extension analysis using RACE revealed a 203-nucleotide 5'UTR and a 123-nucleotide 3’UTR, resulting in a complete 644-nucleotide transcript, including the translated region. The map highlights ([x]) a 75-base pair deletion in asRNA_0067 strategically designed to avoid impacting the *virB2* CDS

The characterization analysis of both transcripts revealed an overlap of 164 nucleotides between asRNA_0067 and the CDS of its antisense gene *virB2*. This finding was further elucidated through the RACE technique, allowing precise determination of the 5’ and 3’ ends of both transcripts. The complete sequence of asRNA_0067 measured 329 nucleotides, with the half length of this sequence overlapping with the *virB2* CDS. Additionally, RACE results for *virB2* showed a downstream extension of 203 nucleotides from the 5’ end and an upstream extension of 123 nucleotides from the 3’ end. Adding the 318 nucleotides from the CDS to the RACE results, we determine that *virB2* transcribes into a 644-nucleotide sequence (Supplementary Information 4). We compiled all the above findings and graphed a scale map based on the Artemis tool (https://www.sanger.ac.uk/tool/artemis/) for visualization. The map depicted in Fig. 4 illustrates a 75-bp deletion within the asRNA_0067 achieved through the isothermal assembly technique. This approach was carefully designed to prevent any impact on the CDS of *virB2* or its hypothetical RBS, considering the possibility of an internal promoter in that region. We conducted sequencing to validate the 75-bp deletion inside the asRNA_0067 (Supplementary Information 5).

### Selective mutation of asRNA_0067 decreased *virB2* transcription under intraphagocytic stressors

**Fig. 5:**
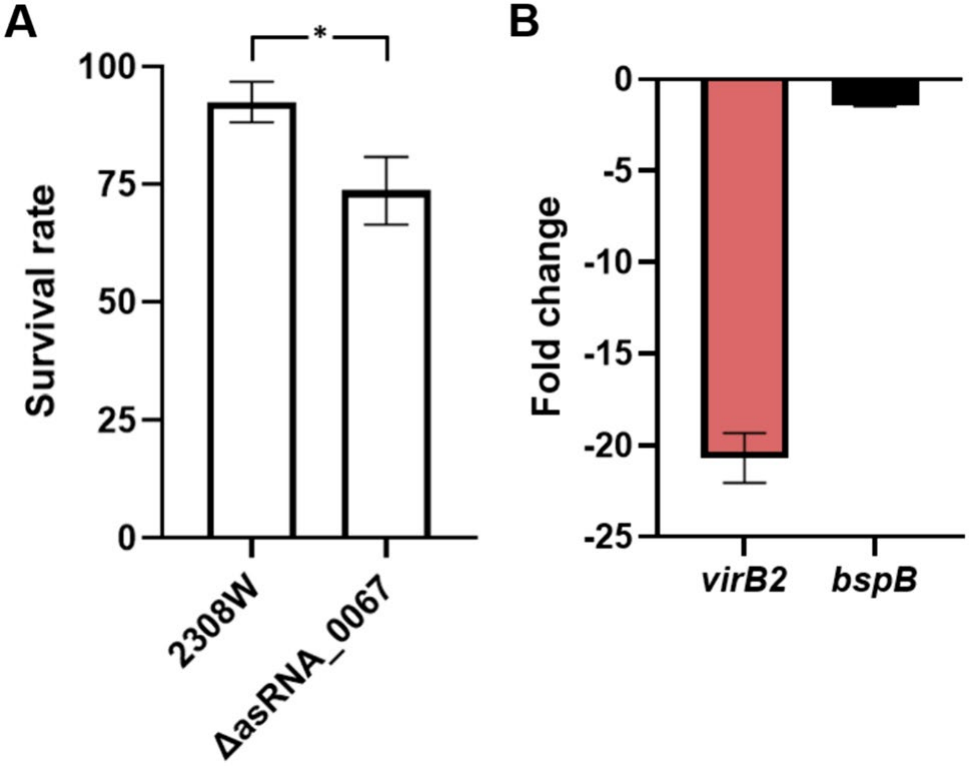
Compared survival and gene expression analysis of *B. abortus* 2308W and its ΔasRNA_0067 deletion mutant at ISM_A pH 4.5 minimal medium. Survival rate (**A**) was calculated as the difference in CFU between 0 and 4 h of incubation. Differential gene expression fold changes (**B**) were obtained by culturing both 2308W and its mutant in ISM_A pH 4.5 minimal medium for 4 h. Values are shown as means of 3 experiments ± standard deviations. *P < 0.05

Since asRNA_0067 showed more robust differential expression under the intraphagocytic stressors, we proceed with the study using the deletion mutant ΔasRNA_0067 constructed by isothermal assembly without affecting CDS or a hypothetical autonomous RBS. The survival rate in the ISM_A1 at 4 h post-inoculation was 93% for 2308W and 74% for ΔasRNA_0067 (Fig. 5A). The growth kinetics were similar between the WT and mutant strains, ensuring that the observed changes were attributed to the intraphagocytic stressors (Supplementary Information 6). The differential expression of two genes selected by sRNA-mRNA predictive interaction analysis, *bspB,* and *virB2*, was evaluated by comparing the parental and deletion strains. The *virB2* gene had the best predictive values because asRNA_0067 is antisense to this *virB* operon gene.

As depicted in Fig. 5B, the mutation of asRNA_0067 results in a 20.6-fold decrease in the expression of the *virB2* gene at the mRNA level compared to the 2308W. Meanwhile, the *bspB* gene, which encodes an effector of T4SS, had a value of − 1.4-fold, which does not exceed the threshold of ± 1.5 fold used to determine minimal differential expression. Although this gene exhibited a high probability (p) of interaction with asRNA_0067, it also had a very high FDR (Table 2), so we assessed *bspB* as a regulatory control. In the deletion mutant ΔasRNA_0067, the absence of the asRNA_0067 was confirmed through RT-PCR (data not shown).

## Discussion

In this study, we identified two RNAs on the complementary strand of the *virB* operon. Notably, asRNA_0067, antisense to *virB2,* displayed elevated expression under intraphagocytic stress conditions characterized by acidity and nutrient deprivation. Additionally, the deletion within asRNA_0067 was associated with a significant reduction in the expression of its antisense gene, *virB2*, compared to the parental strain. To the best of our knowledge, the expression of antisense RNAs to the *virB* operon has never been characterized.

During a comprehensive analysis of open RNA-seq databases, we detected the expression of both antisense RNAs in the wide-genome transcriptome of *B. abortus* 2308W and its mutant Δ*vjbR* under acidity and nutrient deprivation (Kleinman et al. 2017) (Fig. 1A). Interestingly, asRNA_0067 has an increase in transcription counts in mutant Δ*vjbR* compared to the parental strain (Fig. 1C). We found the same antisense RNAs in *B. suis* and *B. microti* under nutrient deprivation and acidification when we used the same RNA-seq analysis methodology employing the dataset PRJNA644280 (de la Garza-García et al. 2021). Then, we validated the presence of both RNAs in our study with RT-qPCR following the established protocol as previously described by Peng et al. (2015) as many other RNAs have been corroborated (King et al. 2022) (Fig. 1B).

The interaction analysis between asRNA_0067 and *virB2* revealed a predictable and statistically significant association, as evidenced by the high p-value and low FDR. Unlike the *bspB* gene, which encodes an effector of T4SS, it displayed an optimal p-value but a high FDR (Table 2). Interestingly, validated interactions between *E. coli* sRNA GcvB and mRNAs have been reported (Sansen et al. 2016), and the FDR values have exceeded 0.05, including values as high as 0.79 with the *livK* gene, a leucine ABC transporter (data not shown). Therefore, we decided to focus on asRNA_0067 and include *virB2* and *bspB* in the differential expression analysis. In addition to those mentioned above in silico analysis, we found that asRNA_0067 exhibited a higher expression level compared to RNA_0069 under intraphagocytic adaptation phase stressors in the same minimal medium MM1 employed in the RNA-seq analysis (Kleinman et al. 2017; Sieira et al. 2010). We added to this essay the minimal medium GEM (Wang et al. 2015), obtaining higher expression of asRNA_0067 (Fig. 2). The RNA_0069 was excluded in this study as it was found to interact with the TonB-dependent copper receptor gene, which may be associated with copper homeostasis and intracellular persistence (Chandrangsu et al. 2017; Achard et al. 2012) and not directly with the adaptation to acidification and nutrient deprivation.

Our investigation also aimed to improve the intraphagocytic simulation media, aligning with the adaptation phase of *Brucella* during intracellular infection. A thorough exploration of pH values, exposure time, and nutrient deprivation degrees guided our efforts to generate optimal conditions for this simulation media (Table 1). Discrepancies in reported pH values led us to evaluate pH 4.5 and 5 in ISM_A1 and ISM_A2, respectively. Existing literature indicates intraphagosomal pH variations between 4.5 reported by Porte et al. (1999) using pH-sensible fluorescent antibodies during the infection of *B. suis* in J774 cells. On the other hand, Bellaire et al. (2005) depicted within a schematic illustration of the intracellular trafficking patterns of opsonized *B. abortus* 2308W inside THP-1 cells that the pH value is near 5 during the adaptative phase when the late endosome is marked with H^+^ v-ATPases. These enzymes generate an acidic environment through proton (H +) pump activity inside endosomes and lysosomes (Cotter et al. 2016; Xia et al. 2019). Other studies about endosome acidification and the endolysosomal system, based on pH dependence of fluorescein fluorescence, determined that late endosomes have a pH between 5 and 5.5 and lysosomes can decrease from 5.4 to 4.5 (Maxfield and Yamashiro 1987; Repnik et al. 2013). Our experimental design considered these variations for a comprehensive perspective essaying under 4.5 and 5 pH values. Despite limited literature on nutrient deprivation in the intracellular infection of *Brucella*, we modified glucose concentration in the GEM minimal medium based on Koobotse et al. (2020), reducing this carbon source from 20 to 2 g/L. Temporal aspects about the exposure to pH 4.5 during *Brucella* resistance to intraphagocytic acidification, aligning with previous observations, indicate that maximum acidification occurs within the first-hour post-infection lasting approximately 4 h (Porte et al. 1999). This timeframe also matches with the *virB* operon peak of expression that is around the fifth-hour post-infection (Sieira 2013).

Our experimental evidence showed that as acidification and nutrient deprivation levels increased, *B. abortus* exhibited higher expression of asRNA_0067 and its antisense gene *virB2*. This proportional upregulation is also evident in the RNA-seq database by Kleinman et al. (2017). The 0.5 pH difference between ISM_A1 (pH 4.5) and ISM_A2 (pH 5) resulted in significant differences in survival rates, as reported by de la Garza-García et al. (2021). Additionally, a nearly twofold increase in the expression of *virB2* and asRNA_0067 was observed in the pH 4.5 medium compared to its pH 5 homolog. Similarly to what has been previously reported, higher expression of *virB* was found in GEM pH 4 compared to the same medium at pH 7 (Wang et al. 2009). The fold change of *bspB* in ISM_A1 (pH 4.5) was 1.6 fold higher compared to that obtained in BB pH 7, just surpassing the threshold for differential expression set at 1.5 ± fold (Vaes et al. 2014, de la Garza-García et al. 2021) (Fig. 3A, B).

Upon standardizing pH across all media employed in this study, we determined that nutrient deprivation levels exerted a more pronounced influence on the expression of asRNA_0067 and *virB2* than the acidification did. In BB pH 4.5, despite having the highest survival rate, the expression of *virB2* and asRNA_0067 was the lowest, indicating that the maximum expression of these two genes is required during more significant nutrient deprivation. Interestingly, in BB pH 4.5, which contains a high amount of nutrients specifically tailored for *Brucella*, the highest level of expression of the effector gene *bspB* was observed (Fig. 3C, D). This expression behavior could be explained by the increased nutrient acquisition possibly acting as a signal for *Brucella* to initiate the secretion of the effector BspB near the endoplasmic reticulum (ER) cisternae, where some *Brucella* cells reach as early as the first 1–2 h post-infection. (Arellano-Reynoso et al. 2005; Celli et al. 2003). There, they acquire nutrients and ER markers for trafficking to the Golgi apparatus, facilitating the biogenesis of the replicative BCV (rBCV) (Celli 2020; Miller et al. 2017). However, further research is needed to understand the nutrient levels during the expression of effectors during the transition from adaptation to replicative phase.

Employing the RACE technique, we obtained the 5’ and 3’ ends of both asRNA_0067 transcripts. The complete sequence of asRNA_0067 spans 329 nucleotides, half overlapping with the *virB2* CDS. Predictive interaction analysis utilizing IntaRNA suggested a substantial interaction spanning approximately 90 nucleotides between asRNA_0067 and *virB2*. This hypothetical interaction hints at a regulatory relationship between these transcripts, potentially influencing the expression of *virB2* as occurs with another cis-encoded RNA, *BsrH,* which regulates the expression of the *hemH* gene (Peng et al. 2015). Experimental validation of this interaction with an overexpression strain further supports its biological relevance. RACE results for *virB2* provided additional insights, revealing a downstream extension of 203 nucleotides from the 5’ end and an upstream extension of 123 nucleotides from the 3’ end. These extensions expand our understanding of the *virB2* transcriptional possible boundaries, offering valuable information for future functional studies. The genetic map (Fig. 4) captures the abovementioned findings, emphasizing a purposeful 75-bp deletion within asRNA_0067 achieved through the isothermal assembly technique (Gibson et al. 2009). This deletion was meticulously designed to avoid any impact on the CDS of *virB2* or its hypothetical Ribosome Binding Site (RBS), taking into account the possible presence of an internal promoter in that region. This mutation also did not affect the predicted binding sites of VjbR inside the *virB* operon (Rivas-Solano et al. 2022) or the interaction site predicted by IntaRNA of the homologous sRNA *in B. melitensis* BSR1141 (Wang et al. 2019) (Supplementary Information 7).VirB2 serves as the main component of the T-pilus structure required to translocate the effectors of *Brucella* from the BCV, crucial for its proper intracellular trafficking and indispensable for survival in macrophages and in the murine model (Den Hartigh et al. 2004; Xiong et al. 2021). Therefore, *virB2* is the gene among the *virB* operon with the highest expression (Supplementary Information 8) to cover all the subunits required to assemble an adequate T4SS. Notably, a considerable reduction in *virB2* expression and a mild decrease in survival rate occurred following the deletion of 75 bp within the asRNA_0067 locus (Fig. 5) without affecting either the protein-coding region or the hypothetical autonomous ribosome binding site (RBS).

This effect of asRNA_0067 deletion mutation over *virB2* expression prompts the exploration of several plausible explanations that may occur simultaneously to enable the complete synthesis of all subunits of *virB2*. The asRNA_0067 transcript may, in the first instance, represent a regulatory asRNA that directly exerts influence over the transcriptional expression of the *virB2* gene. In elucidating a potential regulatory role of asRNA_0067 over *virB2*, mRNA stabilization, and transcriptional termination are the mechanisms through which asRNAs upregulate target genes (Georg and Hess 2011). A reported case of an asRNA that upregulates gene expression occurs through the formation of an RNA-RNA duplex, thus hiding the recognition sites of RNase E and preventing degradation (Stazic et al. 2011). On the other hand, a trans-encoded sRNA that upregulates *virB2* has been reported in *B. melitensis*, though its precise mechanism of action remains indeterminate. This particular RNA is situated within chromosome 1 and relies upon its association with the protective and guiding protein Hfq (Wang et al. 2019). Concurrently, the identification of a 5'UTR of *virB2* through RACE sequencing in this study (Fig. 4) underscores the potential importance of this region. In a phytopathogenic proteobacterium, the ncRNA-associated protein CrsA regulated the *virB* operon by binding to the 5'UTR of *virB7* (Cenens et al. 2020). Chen et al. (2022) demonstrated that 5'UTRs regulate gene expression by controlling translation initiation and stabilizing mRNAs. Therefore, the stability of *virB2* mRNA may be mediated by its 5'UTR and its antisense asRNA_0067. Another plausible scenario resides in a possible autonomous promoter of the *virB2* gene, well-known as internal promoters, which are located inside operons to coordinate the transcription of individual genes (Wang et al. 2021). The *virB2* operon undergoes transcription as a polycistronic mRNA, similar to the process observed in *Agrobacterium tumefaciens*, a rhizobium closely related to *Brucella* (Mossey and Das 2013). However, it is worth noting that changes in the expression of individual genes within polycistronic operons have been reported in other systems, such as the flagellar operon in *Salmonella* Typhimurium (Lawhon et al. 2003). Each explanation raises intriguing questions about the precise function of asRNA_0067 and its role in regulating *virB2*. Future experiments may provide further insights into the underlying mechanism behind this reduction in expression and the functional relationships of asRNA_0067 with the *virB* operon.

### Supplementary Information

Below is the link to the electronic supplementary material.

Supplementary file1 (XLSX 12 KB)

Supplementary file2 (XLSX 35 KB)

Supplementary file3 (DOCX 14 KB)

Supplementary file4 (DOCX 14 KB)

Supplementary file5 (DOCX 30 KB)

Supplementary file6 (DOCX 575 KB)

Supplementary file7 (DOCX 129 KB)

Supplementary file8 (DOCX 28 KB)

Supplementary file9 (DOCX 463 KB)

Supplementary file10 (DOCX 31 KB)

## Data Availability

All survival rates are found in Database 1.xls, while all differential expression analysis data are available in Database 2.xls.
