# Supplementary material for: Increased Brucella abortus asRNA_0067 expression under intraphagocytic stressors is associated with enhanced virB2 transcription
Source: Arch Microbiol. 2024 May 31;206(6):285. doi: 10.1007/s00203-024-03984-8 (PMC11139718; doi:10.1007/s00203-024-03984-8)
Supplement: Supplementary file 3 — Supplementary file3 (DOCX 14 KB) [file 203_2024_3984_MOESM3_ESM.docx]

**Primers for RT-qPCR**

**asRNA_0067**

RNA0067 F 5´ ACA ATG AGG GCC AGC AGT AG 3´

RNA0067 R 5´ AAG CAG ACC GTG CAG AAA TC 3´

**RNA_0069**

RNA0069 F 5´ GGC ATC AGG AAA AGT GGT GT 3´

RNA0069 R 5´ TGA AAT CCA GGC GTT GAG AT 3´

**16S rRNA**

16S rRNA F 5´-TAC CAG CCC TTG ACA TCC-3´

16S rRNA R 5´-TCA TCC CCA CCT TCC TCT-3´

***virB2***

virB2 F 5´-CGC GGA TTC TAC CTC ACC TA-3´

virB2 R 5´-GGA CCA GAT GAT GGC TAT GG-3´

***bspB***

bspB F 5´-ATG CTG GAA AAC CGT CAA AG-3´

bspB R 5´-GTG TTG ATG GAA CCG CTT GT-3´

**Primers for RACE**

**asRNA_0067**

Left67aRACE 5´-GAT TAC GCC AAG CTT **GAT TGC AGC GAT GGA GAC AAT GAG G**-3´ (for RACE 3´)

Right67aRACE 5´-GAT TAC GCC AAG CTT **ACA TTG AGC AAG CAG ACC GTG CAG A**-3´ (for RACE 5´)

Left67aNGSP 5´-GAT TAC GCC AAG CTT **TAG GTG AGG TAG AAT CCG CGA CAG C-3´** (for internal RACE 3´)

Right67aNGSP 5´-GAT TAC GCC AAG CTT **AAA ACC GCT TCC CCC AGC AAG AAG T**-3´ (for internal RACE 5´)

***virB2***

LeftvirB2aRACE 5´-GAT TAC GCC AAG CTT **CCT ACT GCT GGC CCT CAT TGT CTC C**-3´ (for RACE 3´)

RightvirB2aRACE 5´-GAT TAC GCC AAG CTT **GGA ATG CCA TCT TGT AAC CGG ACC A** -3´ (for RACE 5´)

LeftvirB2NGSP 5´-GAT TAC GCC AAG CTT **ATC GCT GCA ATC GAG CCT AAC CTG** -3´ (for internal RACE 3´)

RightvirB2aNGSP 5´-GAT TAC GCC AAG CTT **ATC GAT ACG CCG CTT AGC AAG TCC A** -3´ (for internal RACE 5´)

GSP for obtaining RACE PCR products

NGSP for sequencing the RACE PCR products

^*^ GAT TAC GCC AAG CTT 5´ tail for cloning the RACE product inside the vector pRACE

(SMARTer RACE Kit, Takara Bio Inc., Tokyo, Japan)
