# Supplementary material for: Increased Brucella abortus asRNA_0067 expression under intraphagocytic stressors is associated with enhanced virB2 transcription
Source: Arch Microbiol. 2024 May 31;206(6):285. doi: 10.1007/s00203-024-03984-8 (PMC11139718; doi:10.1007/s00203-024-03984-8)
Supplement: Supplementary file 4 — Supplementary file4 (DOCX 14 KB) [file 203_2024_3984_MOESM4_ESM.docx]

**Primers for Isothermal assembly and mutant verification of asRNA_0067**

| **Template** | **Product (bp)** | **Primers** |
| --- | --- | --- |
| pDS132. | 5142 | pDS132-Iso.F: 5’ CATGTGGAATTCCCATGTCAGCCGTTAAG  pDS132-Iso.R: 5’- GATCCTTTTTAACCCATCACATATACCTGCCG |
| pDS132 | asRNA_0067: 922 | pDS132.F 5’-TGATGGGTTAAAAAGGATCG-3’  pDS132b.R 5’-AACAAGCCAGGGATGTAACG-3' |
| Intergenic region *virB1-virB2*  (Fragment 1) | 441 | pDS132-RNA2_0067-Iso.UF:  5’-ATAGTGAACGGCAGGTATATGTGATGGGTTAAAAAGGATCGCAACCGATGCGCCGCCAGGG  RNA2_0067-Iso.UR:  5’-GATTTGTCTTGCTGACTGCATTTCGGCAC |
| asRNA_0067  (Fragment 2) | 481 | RNA2_0067-Iso.DF:  5’- CCAGCCGACTTGTGCCGAAATGCAGTCAGCAAGACAAATCAGGAATAAAGATCATGAAAACCGCTTCCC  RNA2_0067- pDS132-Iso.DR:  5’-ACACAGGAACACTTAACGGCTGACATGGGAATTCCACATGTGGATCGCCGCGATAACCTGC |
| pDS132 with asRNA_0067  fragment | 922 with both inserts  134 without insert | pDS132.F  5’-TGATGGGTTAAAAAGGATCG-3’  pDS132b.R  5’-AACAAGCCAGGGATGTAACG-3' |
| asRNA_0067  mutatant verification | 881 bp in the mutant  957 bp in the WT | pDS132-RNA2_0067-Iso.UF:  5’-ATAGTGAACGGCAGGTATATGTGATGGGTTAAAAAGGATCGCAACCGATGCGCCGCCAGGG  RNA2_0067- pDS132-Iso.DR:  5’-ACACAGGAACACTTAACGGCTGACATGGGAATTCCACATGTGGATCGCCGCGATAACCTGC |

**Primers for Isothermal assembly and mutant verification of RNA_0069**

| **Template** | **Product (bp)** | **Primers** |
| --- | --- | --- |
| BAB2_0069 Fragment 1 | 467 | pDS132-RNA2_0069-Iso.UF:  5’-ATAGTGAACGGCAGGTATATGTGATGGGTTAAAAAGGATCGGAATGGCAGCAAATCGGCCTCGTGC  RNA2_0069-Iso.UR:  5’-TCAGCGCCGACGCAGGACGGA |
| RNA2_0069 Fragment 2 | 399 | RNA2_0069-Iso.DF:  5’TCCGAGCAAGGAAGTCCTTTCCGTCCTGCGTCGGCGCTGAGATGTCTGCCAATTATGTAGAGCGAT  RNA2_0069-pDS132-Iso.DR:  5’-ACACAGGAACACTTAACGGCTGACATGGGAATTCCACATGCAGAGAATGGACGATTATTATGATAGCCT |
| pDS132 with the fragment RNA_0069 | 866 with both inserts  121 without insert. | pDS132.F  5’-TGATGGGTTAAAAAGGATCG-3’  pDS132b.R  5’-aacaagccagggatgtaacg-3' |
| RNA_0069 mutant verification | 917 bp in the mutant  992 bp in the WT | pDS132-RNA2_0067-Iso.UF:  5’-ATAGTGAACGGCAGGTATATGTGATGGGTTAAAAAGGATCGCAACCGATGCGCCGCCAGGG  RNA2_0067- pDS132-Iso.DR:  5’-ACACAGGAACACTTAACGGCTGACATGGGAATTCCACATGTGGATCGCCGCGATAACCTGC |
