# Supplementary material for: Increased Brucella abortus asRNA_0067 expression under intraphagocytic stressors is associated with enhanced virB2 transcription
Source: Arch Microbiol. 2024 May 31;206(6):285. doi: 10.1007/s00203-024-03984-8 (PMC11139718; doi:10.1007/s00203-024-03984-8)
Supplement: Supplementary file 5 — Supplementary file5 (DOCX 30 KB) [file 203_2024_3984_MOESM5_ESM.docx]

**Comparison of survival rate and fold change expression between ISM_R pH 6.5 and pH 4.5**
