# Supplementary material for: Increased Brucella abortus asRNA_0067 expression under intraphagocytic stressors is associated with enhanced virB2 transcription
Source: Arch Microbiol. 2024 May 31;206(6):285. doi: 10.1007/s00203-024-03984-8 (PMC11139718; doi:10.1007/s00203-024-03984-8)
Supplement: Supplementary file 6 — Supplementary file6 (DOCX 575 KB) [file 203_2024_3984_MOESM6_ESM.docx]

**RACE amplification and sequencing of asRNA_0067 and *virB2***


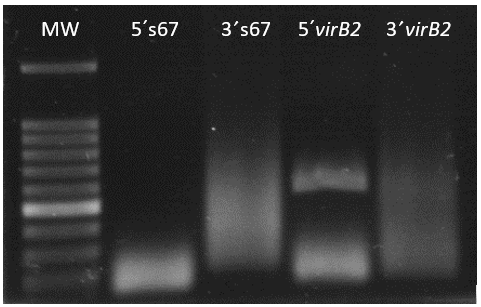


For *virB2* 5'RACE we only purified and sequenced the smaller band.

For both 3'RACE we purified the complete product.

All products were sequenced using internal primers (NGSP).

“Some SMARTer RACE reactions produce very complex patterns of bands that appear almost as smears” (SMARTer® RACE 5'/3' Kit User Manual).

**Sequence of RACE 3´ asRNA_0067 (s67)**


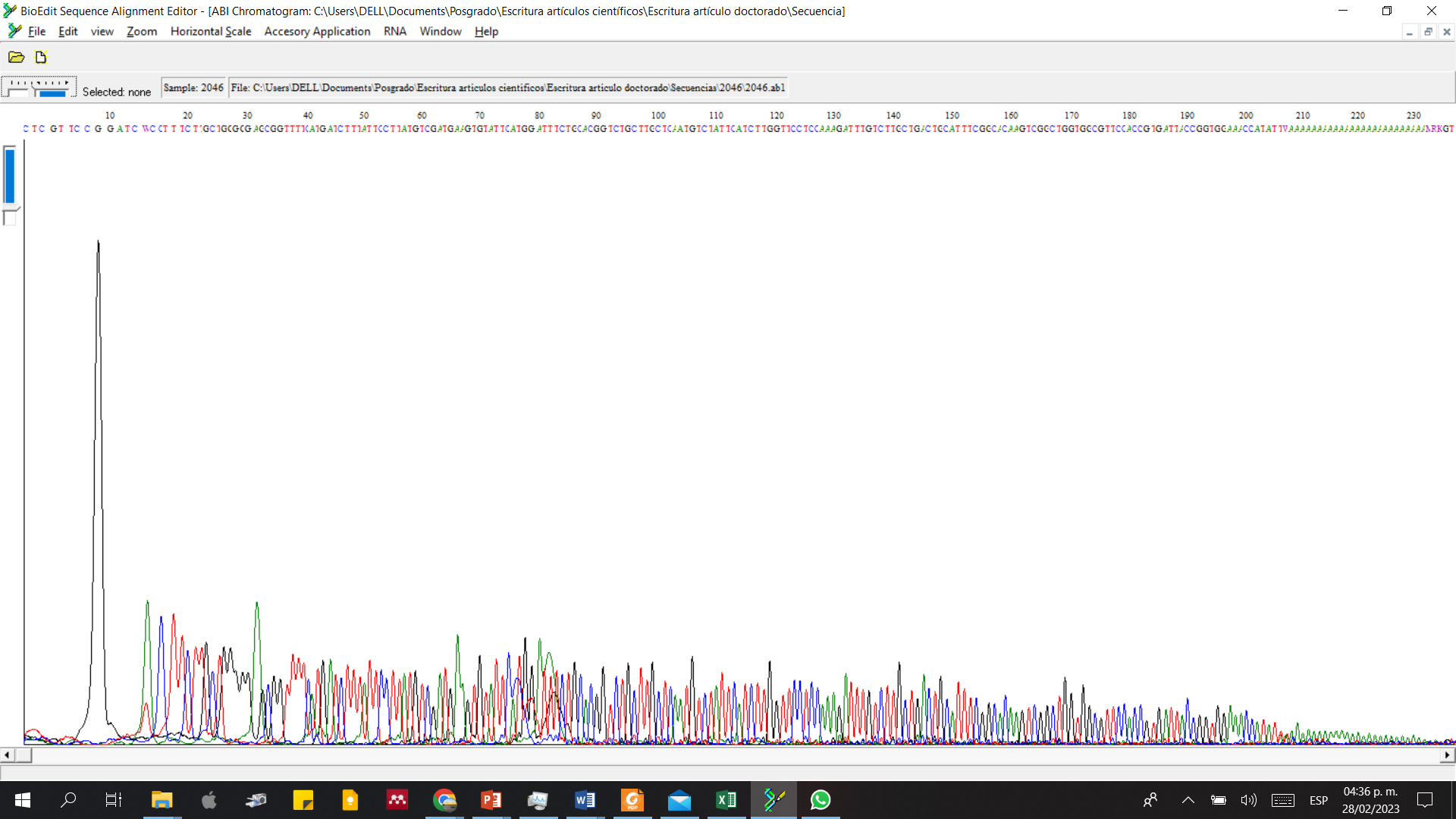
 (28 adenines from polyadenylation)

Successful sequencing is indicated by the presence of the poly(A) tail in the 3' RACE products.

CTTGCTGGGGGAAGCGGTTTTCATGATCTTTATTCCTTATGTCGATGAAGTGTATTCATGGATTTCTGCACGGTCTGCTTGCTCAATGTCTATTCATCTTGGTTCCTCCAAAGATTTGTCTTGCTGACTGCATTTCGGCACAAGTCGGCTGGTGGCGTTCCACCGTGATTACCGGTGGAAACCATATTTAAAAAAAAAAAAAAAAAAAAAAAAAAAA


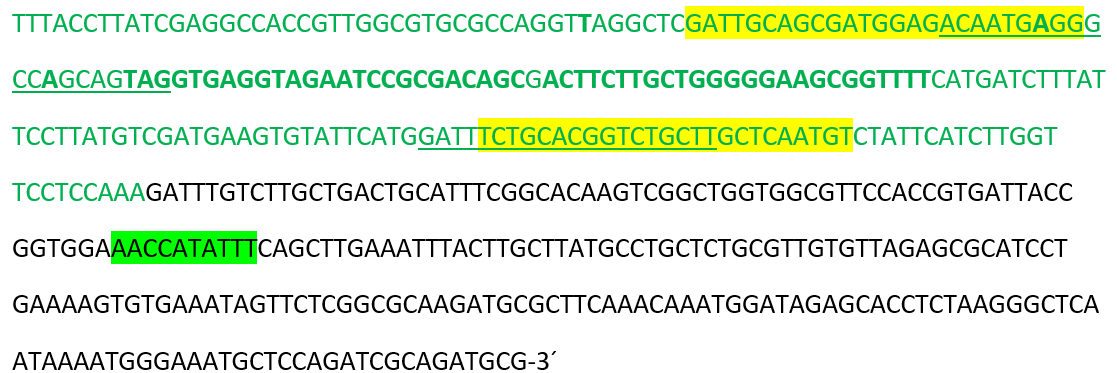


**RACE and RNA-seq based complete asRNA_0067 sequence**

RNA-seq-predicted_5´-CTTAGCAAGTCCAGCACTTTTTGCATGCTTGTATTTACCTTATCGAGGCCACCGTTGGCGTGCGCCAGGTTAGGCTCGATTGCAGCGATGGAGACAATGAGGGCCAGCAGTAGGTGAGGTAGAATCCGCGACAGCGACTTCTTGCTGGGGGAAGCGGTTTTCATGATCTTTATTCCTTATGTCGATGAAGTGTATTCATGGATTTCTGCACGGTCTGCTTGCTCAATGTCTATTCATCTTGGTTCCTCCAAAGATTTGTCTTGCTGACTGCATTTCGGCACAAGTCGGCTGGTGGCGTTCCACCGTGATTACCGGTGGAAACCATATTT- RACE 3´

**Sequence of RACE 5´ *virB2***


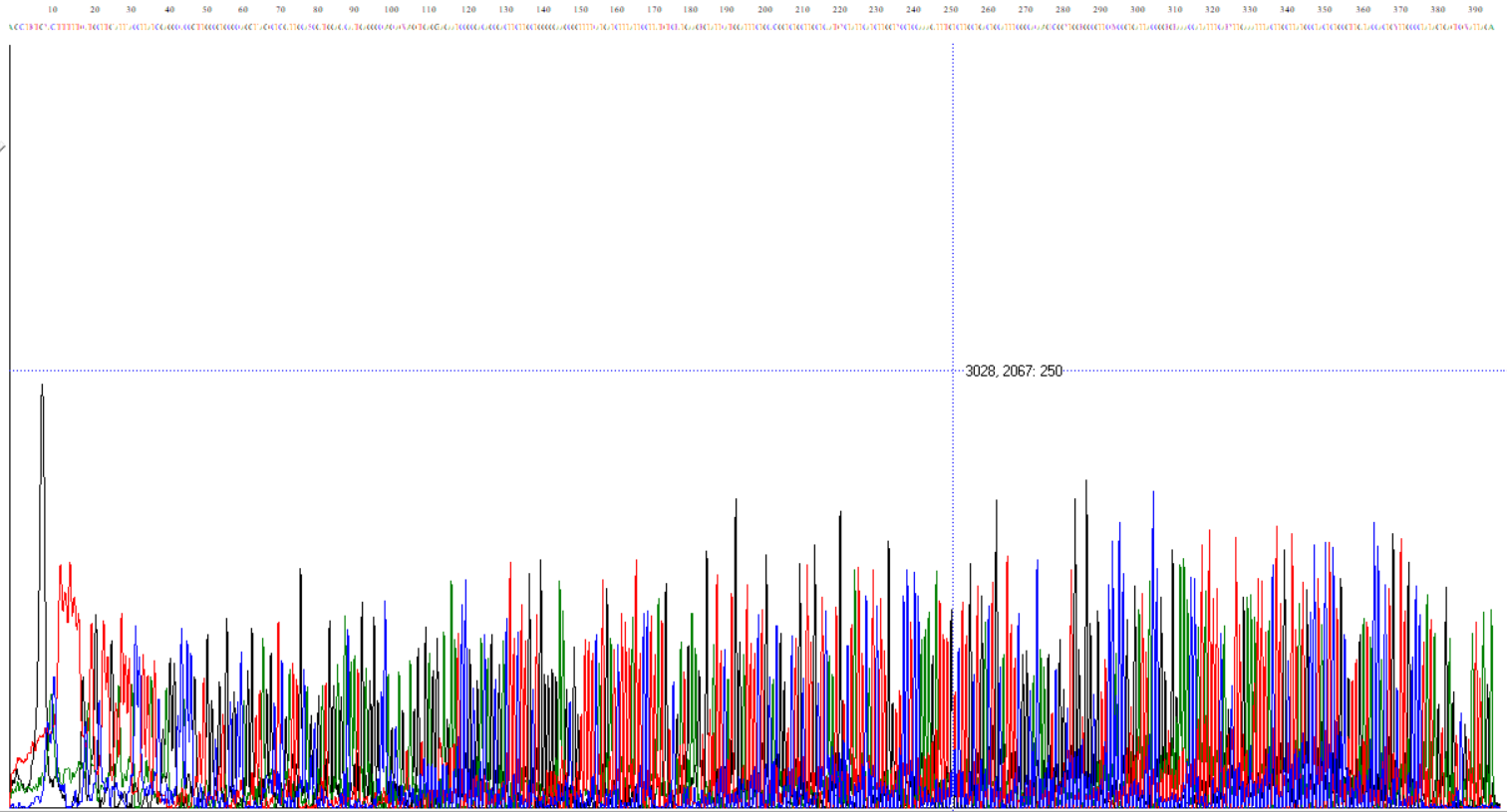


GCTTGTATTTACCTTATCGAGGCCACCGTTGGCGTGCGCCAGGTTAGGCTCGATTGCAGCGATGGAGACAATGAGGGCCAGCAGTAGGTGAGGAGAATCCGCGACAGCGACTTCTTGCTGGGGGAAGCGGTTTTCATGATCTTTATTCCTTATGTCGATGAAGTGTATTCATGGATTTCTGCACGGTCTGCTTGCTCAATGTCTATTCATCTTGGTTCCTCCAAAGATTTGTCTTGCTGACTGCATTTCGGCACAAGTCGGCTGGTGGCGTTCCACCGTGATTACCGGTGGAAACCATATTTCAGCTTGAAATTTACTTGCTTATGCCTGCTCTGCGTTG

(Reverse and complementary)

CAACGCAGAGCAGGCATAAGCAAGTAAATTTCAAGCTGAAATATGGTTTCCACCGGTAATCACGGTGGAACGCCACCAGCCGACTTGTGCCGAAATGCAGTCAGCAAGACAAATCTTTGGAGGAACCAAGATGAATAGACATTGAGCAAGCAGACCGTGCAGAAATCCATGAATACACTTCATCGACATAAGGAATAAAGATCATGAAAACCGCTTCCCCCAGCAAGAAGTCGCTGTCGCGGATTCTCCTCACCTACTGCTGGCCCTCATTGTCTCCATCGCTGCAATCGAGCCTAACCTGGCGCACGCCAACGGTGGCCTCGATAAGGTAAATACAAGC

**Sequence of RACE 3´*virB2***


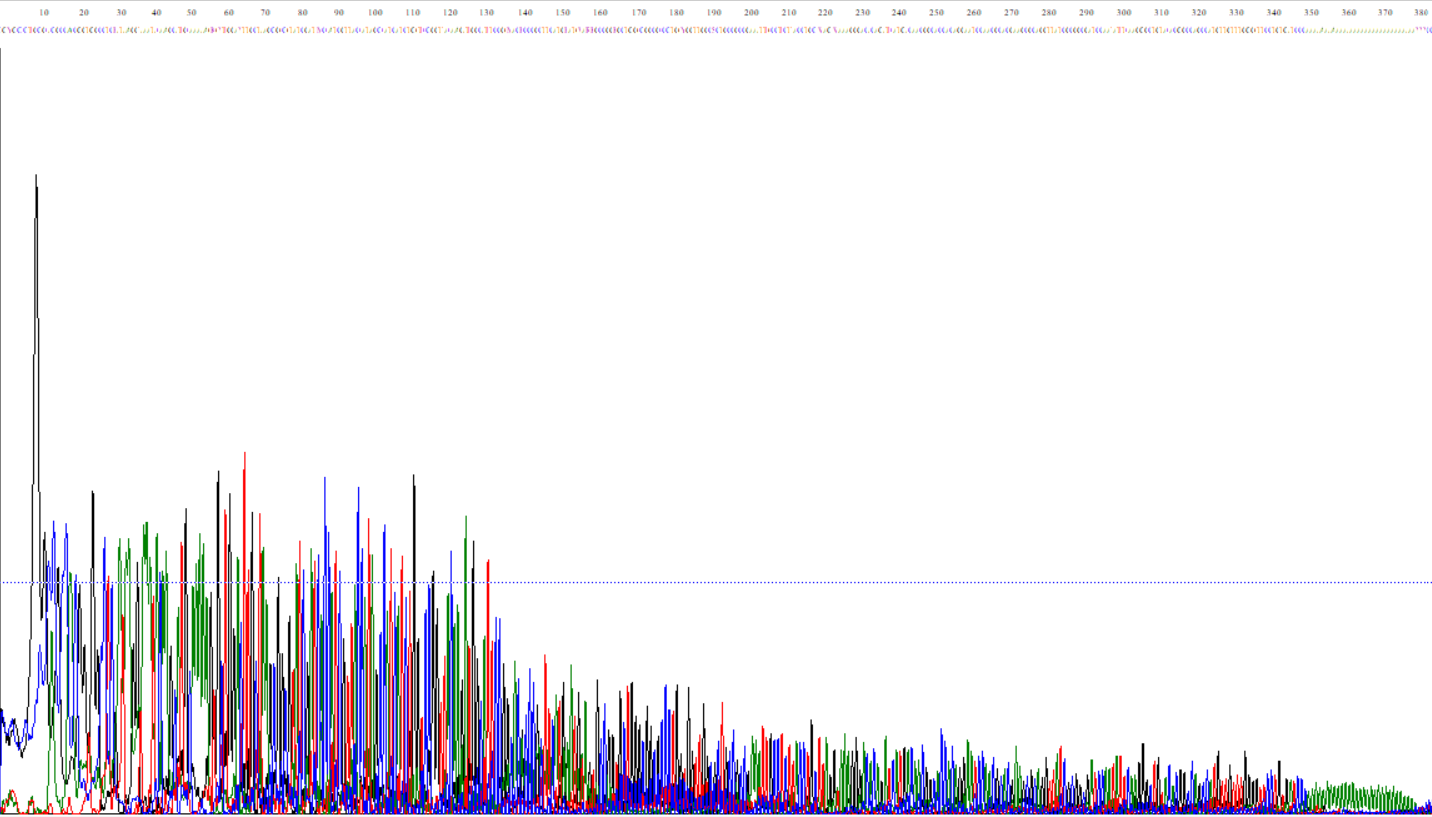
 (29 adenines from polyadenylation)

Successful sequencing is indicated by the presence of the poly(A) tail in the 3' RACE products.

AGCGGCGTATCGATCACCATCGTTACCATAGCCATCATCTGGTCCGGTTACAAGATGGCATTCCGGCACGCCCGCTTCATGGATGTAGTGCCGGTGCTGGGCGGCGCCCTGGTGGTTGGCGCTGCCGCCGAAATTGCCTCTTACCTGCTTAGGTAAAGGGACACAGATCATGACAACGGCACCACAGGAATCCAACGCACGAAGCGCAGGTTATCGCGGCGATCCAATATTCAAGGGCTGTACACGGCCAGCCATGTTGTTTGGGGTTCCTGTGATCCCAAAAAAAAAAAAAAAAAAAAAAAAAAAAA

ACGCATCTGCGATCTGGAGCATTTCCCATTTTATTGAGCCCTTAGAGGTGCTCTATCCATTTGTTTGAAGCGCATCTTGCGCCGAGAACTATTTCACACTTTTCAGGATGCGCTCTAACACAACGCAGAGCAGGCATAAGCAAGTAAATTTCAAGCTGAAATATGGTTTCCACCGGTAATCACGGTGGAACGCCACCAGCCGACTTGTGCCGAAATGCAGTCAGCAAGACAAATCTTTGGAGGAACCAAGATGAATAGACATTGAGCAAGCAGACCGTGCAGAAATCCATGAATACACTTCATCGACATAAGGAATAAAGATCATGAAAACCGCTTCCCCCAGCAAGAAGTCGCTGTCGCGGATTCTACCTCACCTACTGCTGGCCCTCATTGTCTCCATCGCTGCAATCGAGCCTAACCTGGCGCACGCCAACGGTGGCCTCGATAAGGTAAATACAAGCATGCAAAAAGTGCTGGACTTGCTAAGCGGCGTATCGATCACCATCGTTACCATAGCCATCATCTGGTCCGGTTACAAGATGGCATTCCGGCACGCCCGCTTCATGGATGTAGTGCCGGTGCTGGGCGGCGCCCTGGTGGTTGGCGCTGCCGCCGAAATTGCCTCTTACCTGCTTAGGTAAAGGGACACAGATCATGACAACGGCACCACAGGAATCCAACGCACGAAGCGCAGGTTATCGCGGCGATCCAATATTCAAGGGCTGTACACGGCCAGCCATGTTGTTTGGGGTTCCTGTGATCCCGCTTGTCATCGTTGGCGGCAGCATCGTTCTCTTATCGGTCTGGATTTCCATGTTCATATTGCCGCTGATCGTACCAATCGTGCTGGTCATGCGGCAGATCACGCAGACTGACGATCAGATGTTC

**RACE based complete *virB2* sequence**

**(203 nt of 5´UTR)**

**RACE 5´-**CAACGCAGAGCAGGCATAAGCAAGTAAATTTCAAGCTGAAATATGGTTTCCACCGGTAATCACGGTGGAACGCCACCAGCCGACTTGTGCCGAAATGCAGTCAGCAAGACAAATCTTTGGAGGAACCAAGATGAATAGACATTGAGCAAGCAGACCGTGCAGAAATCCATGAATACACTTCATCGACATAAGGAATAAAGATCATGAAAACCGCTTCCCCCAGCAAGAAGTCGCTGTCGCGGATTCTACCTCACCTACTGCTGGCCCTCATTGTCTCCATCGCTGCAATCGAGCCTAACCTGGCGCACGCCAACGGTGGCCTCGATAAGGTAAATACAAGCATGCAAAAAGTGCTGGACTTGCTAAGCGGCGTATCGATCACCATCGTTACCATAGCCATCATCTGGTCCGGTTACAAGATGGCATTCCGGCACGCCCGCTTCATGGATGTAGTGCCGGTGCTGGGCGGCGCCCTGGTGGTTGGCGCTGCCGCCGAAATTGCCTCTTACCTGCTTAGGTAAAGGGACACAGATCATGACAACGGCACCACAGGAATCCAACGCACGAAGCGCAGGTTATCGCGGCGATCCAATATTCAAGGGCTGTACACGGCCAGCCATGTTGTTTGGGGTTCCTGTGATCCC**-RACE 3´**

**(123 nt of 3´UTR)**
