# Supplementary material for: Increased Brucella abortus asRNA_0067 expression under intraphagocytic stressors is associated with enhanced virB2 transcription
Source: Arch Microbiol. 2024 May 31;206(6):285. doi: 10.1007/s00203-024-03984-8 (PMC11139718; doi:10.1007/s00203-024-03984-8)

***virB2* gene of *Brucella abortus* 2308 (190 nt upstream)**

GCATAAGCAAGTAAATTTCAAGCTGAAATATGGTTTCCACCGGTAATCACGGTGGAACGCCACCAGCCGACTTGTGCCGAAATGCAGTCA**G**CAAGACAAATC***~~TTTGGAGGAACCAAGATGAATAGACATTGAGCAAGCAGACCGTGCAGAAATCCATGAATACACTTCATCGACATA~~AGGAA****TAAAGATC****A****TGAAAACCGCTTCCCCCAGCAAGAAGTCGCTGTCGCGGATTCTACCTCACCTACTGCTGGCCCTCATTGTCTCCATCGCTGCAATCGAGCCTAACCTGGCGCACGCCAACGGTGGCCTCGATA****AGGTAAA****TACAAGCATGCAAAAAGTGCTGGACTTGCTAAGCGGCGTATCGATCACCATCGTTACCATAGCCATCATCTGGTCCGGTTACAAGATGGCATTCCGGCACGCCCGCTTCATGGATGTAGTGCCGGTGCTGGGCGGCGCCCTGGTGGTTGGCGCTGCCGCCGAAATTGCCTCTTACCTGCTTAGGTAA*AGGGACACAGATCATGACAACGGCACCACAGGAATCCAACGCACGAAGCGCAGGTTATCGCGGCGATCCA

***virB2* gene of *Brucella abortus* 2308 ΔasRNA_0067**

**depicting the 75 bp deleted by isothermal assembly mutation**

GCATAAGCAAGTAAATTTCAAGCTGAAATATGGTTTCCACCGGTAATCACGGTGGAACGCCACCAGCCGACTTGTGCCGAAATGCAGTCAGCAAGACAAATC___________________________________________________________________________AGGAATAAAGATC*ATGAAAACCGCTTCCCCCAGCAAGAAGTCGCTGTCGCGGATTCTACCTCACCTACTGCTGGCCCTCATTGTCTCCATCGCTGCAATCGAGCCTAACCTGGCGCACGCCAACGGTGGCCTCGATAAGGTAAATACAAGCATGCAAAAAGTGCTGGACTTGCTAAGCGGCGTATCGATCACCATCGTTACCATAGCCATCATCTGGTCCGGTTACAAGATGGCATTCCGGCACGCCCGCTTCATGGATGTAGTGCCGGTGCTGGGCGGCGCCCTGGTGGTTGGCGCTGCCGCCGAAATTGCCTCTTACCTGCTTAGGTAA*AGGGACACAGATCATGACAACGGCACCACAGGAATCCAACGCACGAAGCGCAGGTTATCGCGGCGATCCA

**Validation of the 75 bp deleted in the ΔasRNA_0067 by sequencing**


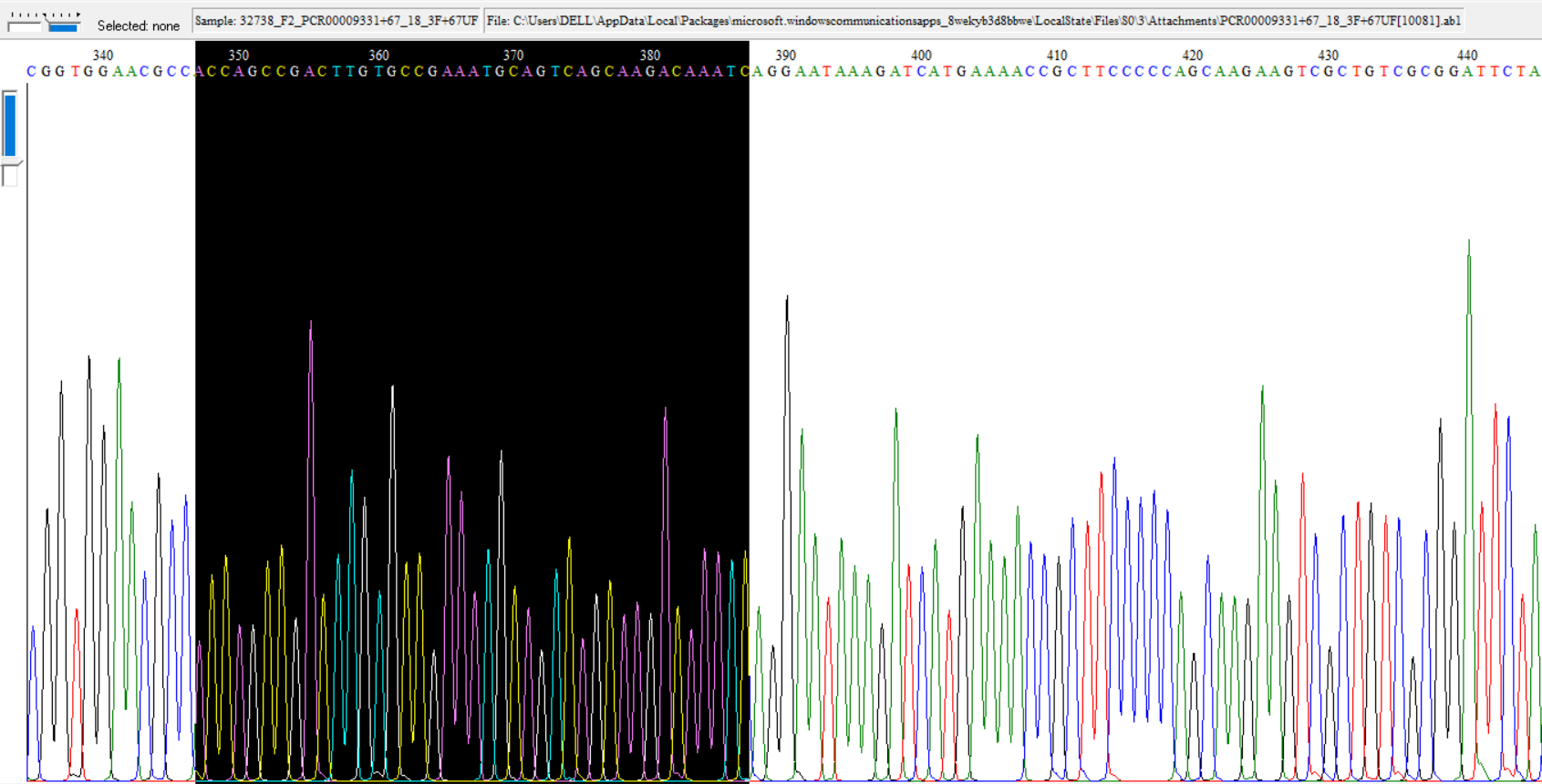

Supplement: Supplementary file 7 — Supplementary file7 (DOCX 129 KB) [file 203_2024_3984_MOESM7_ESM.docx]
