# Supplementary material for: Increased Brucella abortus asRNA_0067 expression under intraphagocytic stressors is associated with enhanced virB2 transcription
Source: Arch Microbiol. 2024 May 31;206(6):285. doi: 10.1007/s00203-024-03984-8 (PMC11139718; doi:10.1007/s00203-024-03984-8)
Supplement: Supplementary file 8 — Supplementary file8 (DOCX 28 KB) [file 203_2024_3984_MOESM8_ESM.docx]

**Basal growth kinetics of *Brucella abortus* 2308W and its mutant ΔasRNA_0067**

The basal growth kinetics of *Brucella abortus* 2308 and its mutant ΔasRNA_0067 were evaluated to assess their growth patterns under normal conditions in the Brucella Broth at pH 7. The results indicate similar growth rates between the wild-type and mutant strains, suggesting that any observed changes in subsequent experiments can be attributed to specific stressors or experimental conditions rather than inherent differences in baseline growth.
