# Supplementary material for: Increased Brucella abortus asRNA_0067 expression under intraphagocytic stressors is associated with enhanced virB2 transcription
Source: Arch Microbiol. 2024 May 31;206(6):285. doi: 10.1007/s00203-024-03984-8 (PMC11139718; doi:10.1007/s00203-024-03984-8)

**Sequence of virB1, virB2, asRNA_0067 and its 75-bp deletion assessed in this study**

ACACTACCCAATAATGACCGATATCGCTGATCTATAATTAAGGCTATCATAATAATCGTCCATTCTCTGAGAAGGAGACGATCCT**ATG**GTGCCATTCCTTGTCCTCGCGCAACAATGCGCACCGACTGTTGCACCTCAGACTATGGCAGCAATCGTGCAGGTCGAGT**CGGGCTTCAATCCT TATGCAATAGGCGTCGTTGGTGGGCGGTTGGTC**CGTCAACCCGTTT**CCCTTGATGAAGCAATCACGACAGCACAGTCACTGGAAGCCAAAGGCTGGAATTTCTCTTTGGGTATTGCTCAAGTCA**ACAGGTACAATCTGCCGAAATATGGCAGCACCTACGCACAAGCGTTCGACCCCTGCAAGAACCTGAAGATGGGATCCAAGATCCTTGAAGACTGCTACCGTCGGGCCATCGTGAAGATGCCCGGTCAGGAACAAGGCGCGCTTCGCGCCGCATTCTCCTGTTACTACGCCGGCAACTTTACGGGCGGCTTCAAGACGAAGCCCGGCAGTCCCAGCTACGTGCAGAAGGTCGTGGCAAGCGCCGACGTGACCACAAAGCCGATTGTTGTCGTGCCCATGATCCGGAAAACGCCGGATGCGGCGGCAGCAGTAGCTGCCCCAGTAAAAAAACGACAGCCGGCTGATCGTAATTCTGTTCTTGTCGATCTGCATCCATCATCGCAGTCGATGCCAGCCACCGGCACGGCGAACGCGCCTGTAAGGCTGAAGACAGAGCAGCCGGCGACAACCGATGCGCCGCCAGGGAAGGATAATACGGACGGCGTAGTTGTTTTCTAACCCATCATCCGATCAGGCACGCATATAAAATTGATGATGGTGGAGGGTCCGAACAGGACTGGGAAGGTTATAGCGGCGGGCGGCGACGATAGGAGGCAGTCGCACACCATAGACGCATCTGCGATCTGGAGCATTTCCCATTTTATTGAGCCCTTAGAGGTGCTCTATCCATTTGTTTGAAGCGCATCTTGCGCCGAGAACTATTTCACACTTTTCAGGATGCGCTCTAACACAACGCAGAGCAGGCATAAGCAAGTAAATTTCAAGCTGAAATATGGTTTCCACCGGTAATCACGGTGGAACGCCACCAGCCGACTTGTGCCGAAATGCAGTCAGCAAGACAAATC**~~TTTGGAGGAACCAAGATGAATAGACATTGAGCAAGCAGACCGTGCAGAAATCCATGAATACACTTCATCGACATA~~AGGAA**TAAAGATC**ATG**AAAACCGCTTCCCCCAGCAAGAAGTCGCTGTCGCGGATTCTACCTCACCTACTGCTGGCCCTCATTGTCTCCATCGCTGCAATCGAGCCTAACCTGGCGCACGCCAACGGTGGCCTCGATAAGGTAAATACAAGCATGCAAAAAGTGCTGGACTTGCTAAGCGGCGTATCGATCACCATCGTTACCATAGCCATCATCTGGTCCGGTTACAAGATGGCATTCCGGCACGCCCGCTTCATGGATGTAGTGCCGGTGCTGGGCGGCGCCCTGGTGGTTGGCGCTGCCGCCGAAATTGCCTCTTACCTGCTTAGGTAAAGGGACACAGATCATGACAACGGCACCACAGGAATCCAACGCACGAAGCGCAGGTTATCGCGGCGATCCAATATTCAAGGGCTGTACACGGCCAGCCATGTTGTTTGGGGTTCCTGTGATCCCGCTTGTCATCGTTGGCGGCAGCATCGTTCTCTTATCG**GTCTGGATTTCCAT**GTTCATATTGCC

sRNA BSR1141 [2] predicted interaction

**5’** end ☺

*virB2*

**5’ end**

as67

hVjbR

BS [1]

hVjbR

BS [1]

**3’** ☺

*virB2*

**3’** ☺

as67

hRBS

**~~75 bp-del.~~**

asRNA_0067

VirB2

VirB1

RBS


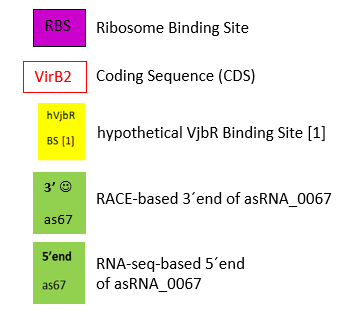

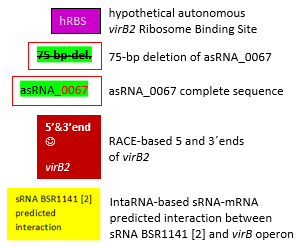


Reference mentioned in this chart:

[1] Rivas-Solano, O., Van der Henst, M., Castillo-Zeledón, A., Suárez-Esquivel, M., Muñoz-Vargas, L., Capitan-Barrios, Z., ... & Guzmán-Verri, C. (2022). The regulon of *Brucella abortus* two-component system BvrR/BvrS reveals the coordination of metabolic pathways required for intracellular life. *Plos one*, *17*(9), e0274397.


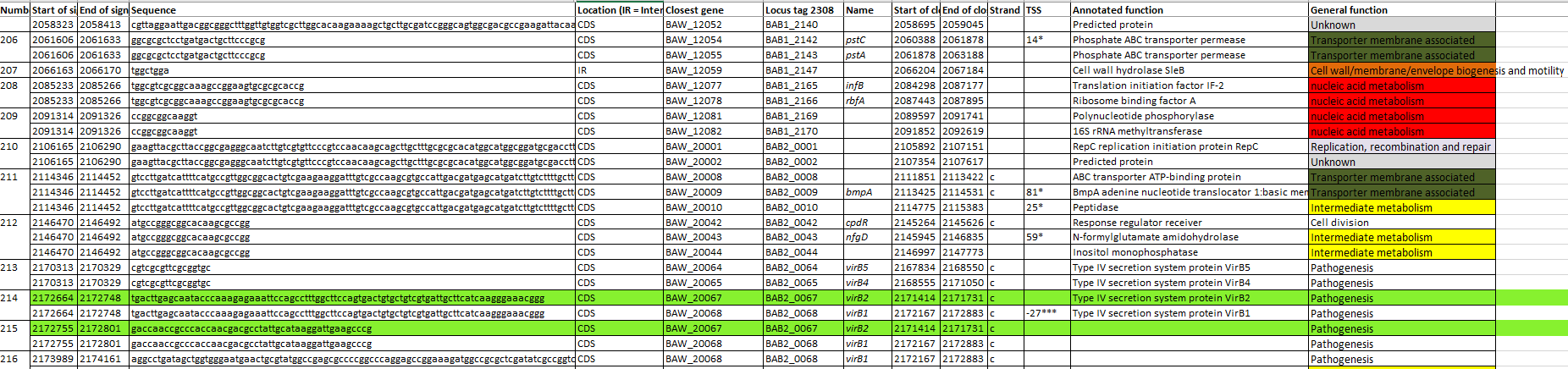

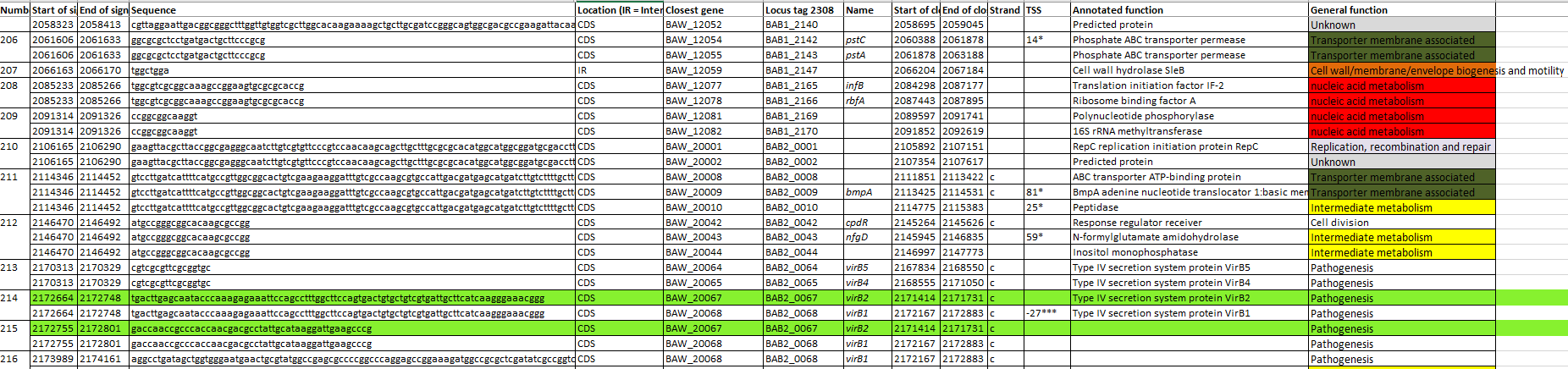


Reference mentioned in this chart:

[2] Wang, Y., Ke, Y., Duan, C., Ma, X., Hao, Q., Song, L., ... & Chen, Z. (2019). A small non-coding RNA facilitates Brucella melitensis intracellular survival by regulating the expression of virulence factor. *International Journal of Medical Microbiology*, *309*(3-4), 225-231


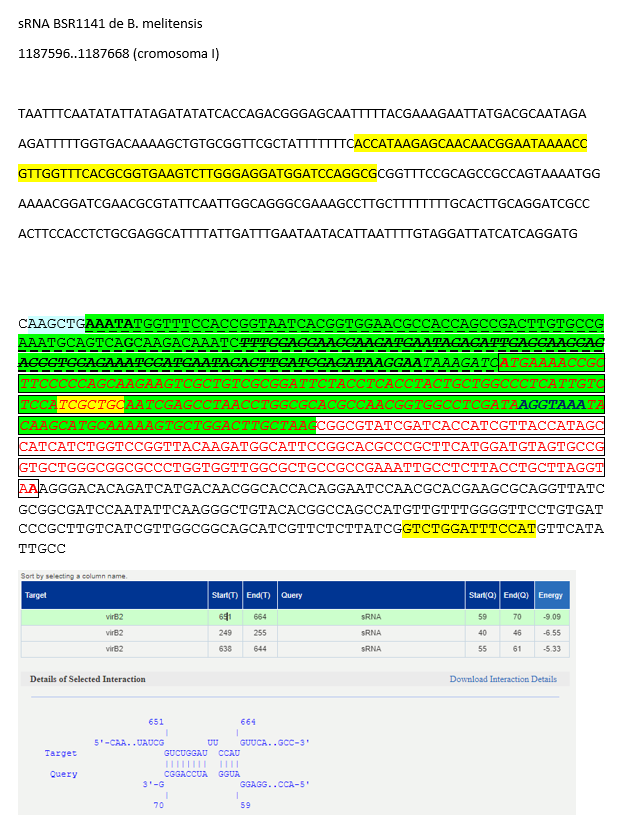

Supplement: Supplementary file 9 — Supplementary file9 (DOCX 463 KB) [file 203_2024_3984_MOESM9_ESM.docx]
