# Supplementary material for: Increased Brucella abortus asRNA_0067 expression under intraphagocytic stressors is associated with enhanced virB2 transcription
Source: Arch Microbiol. 2024 May 31;206(6):285. doi: 10.1007/s00203-024-03984-8 (PMC11139718; doi:10.1007/s00203-024-03984-8)
Supplement: Supplementary file 10 — Supplementary file10 (DOCX 31 KB) [file 203_2024_3984_MOESM10_ESM.docx]

***virB2* is the most transcribed gene within the *virB* operon**

The depicted graph relies on data extracted from the RNA-seq analysis conducted by Kleinman et al. 2017.

Differential expression of all the *virB* operon genes of *Brucella abortus* 2308 when compared to its Δ*vjbR* mutant. The culture where incubated in the minimal medium MM1 at pH 5.5 for 3 h.

Kleinman, C. L., Sycz, G., Bonomi, H. R., Rodríguez, R. M., Zorreguieta, A., & Sieira, R. (2017). ChIP-seq analysis of the LuxR-type regulator VjbR reveals novel insights into the *Brucella* virulence gene expression network. *Nucleic acids research*, *45*(10), 5757-5769. <https://doi.org/10.1093/nar/gkx165>
